# Dissection of Immune Profiles in Microsatellite Stable and Low Microsatellite Instability Colon Adenocarcinoma by Multiomics Data Analysis

**DOI:** 10.1155/2022/8588164

**Published:** 2022-04-15

**Authors:** Tao Yang, Jiali Lei, Qiushi Feng, Dandan Song, Xiaosheng Wang

**Affiliations:** ^1^Biomedical Informatics Research Lab, School of Basic Medicine and Clinical Pharmacy, China Pharmaceutical University, Nanjing 211198, China; ^2^Cancer Genomics Research Center, School of Basic Medicine and Clinical Pharmacy, China Pharmaceutical University, Nanjing 211198, China; ^3^Big Data Research Institute, China Pharmaceutical University, Nanjing 211198, China

## Abstract

**Background:**

Although microsatellite instability (MSI) is an indicator for active immunotherapy response, only 15% of colon adenocarcinoma (COAD) patients are with MSI. An investigation into the immune profiles in low MSI (MSI-L) and microsatellite stable (MSS) COAD remains lacking, whereas such exploration may provide new insights into COAD immunity.

**Methods:**

We hierarchically clustered MSI-L/MSS COAD based on the enrichment levels of 28 immune signatures to identify its immune-specific subtypes. We also comprehensively compared molecular and clinicopathologic profiles among these subtypes.

**Results:**

We identified three immune subtypes of MSI-L/MSS COAD (IM-H, IM-M, and IM-L), which had high, medium, and low immune signature scores, respectively. We demonstrated that this subtyping method was reproducible and predictable by analyzing five different datasets, including four bulk tumor datasets and one single-cell dataset. IM-H was characterized by high immunity, high stemness, strong potential of proliferation, invasion and metastasis, epithelial-mesenchymal transition, elevated expression of oncogenic pathways, low tumor purity, low intratumor heterogeneity (ITH), genomic instability, inferior response to chemotherapy, and unfavorable prognosis. IM-M was characterized by the highest ratio of immunostimulatory to immunosuppressive signatures, the best response to chemotherapy, and favorable prognosis. IM-L was characterized by low immunity, high tumor purity, high ITH, and genomic stability.

**Conclusion:**

The immune-specific subtyping of MSI-L/MSS COAD may provide new insights into the tumor immunity as well as clinical implications for immunotherapy of the COAD patients who lack MSI.

## 1. Introduction

Colorectal cancer (CRC), including colon cancer and rectal cancer, is the third most common cancer and the fourth leading cause of cancer deaths worldwide [1]. Although early-stage CRCs are often curative by surgical resection alone, late-stage CRCs have a poor prognosis due to recurrence or metastasis [2]. In CRC, colon cancer or colon adenocarcinoma (COAD) is more common than rectal cancer [3]. Previous studies have shown that COAD is highly heterogeneous in molecular profiles [4, 5]. For example, the TCGA Research Network identified three molecular subtypes of COAD, including chromosomal instability (CIN), microsatellite instability (MSI), and CpG island methylator phenotype (CIMP) [6]. MSI, resulting from inactivation of the mismatch repair (MMR) system by either MMR gene mutations or hypermethylation of the MLH1 promoter, occurs in around 15% of colon cancers [7]. Based on the MSI status, COAD can be divided into three subgroups: MSI-H (high-frequency microsatellite instability), MSI-L (low-frequency microsatellite instability), and MSS (microsatellite stable). Major clinicopathologic and molecular features show no significant difference between MSI-L and MSS tumors, although they are significantly different between MSI-H and MSI-L/MSS tumors [8]. MSI-H tumors are characterized by the strong lymphocyte infiltration, high tumor mutation burden (TMB), and high expression of immune checkpoint molecules, e.g., PD-L1 [9], and are thus more responsive to immunotherapies. As a result, MSI-H COAD patients have a more favorable prognosis than MSI-L/MSS patients [10].

Antitumor immunotherapies have recently been shown to be effective in treating various cancers [11]. Particularly, immune checkpoint inhibitors (ICIs) targeting cytotoxic T lymphocyte-associated antigen 4 (CTLA-4) and the programmed cell death protein 1 pathway (PD-1/PD-L1) have demonstrated successes in treatment of many refractory malignancies [12]. Nevertheless, currently, only a subset of cancer patients respond to ICIs [13]. To improve the response rate to ICIs in cancer patients, certain biomarkers have been identified, including *PD-L1* expression [14], TMB [15], and DNA damage repair deficiency or MSI [16]. In fact, besides its predictive value in the response to classic therapy with 5-FU [17], MSI is an indicator for the active response to immunotherapy [16]. Notably, the US Food and Drug Administration (FDA) have approved ICIs for treating solid tumors with high MSI [18]. Nevertheless, the immunotherapeutic efficiency for the majority of colon cancers, which are MSI-L/MSS, remains unclear or unexplored. Therefore, it is crucial to stratify MSI-L/MSS COAD patients responsive to immunotherapies.

It has been shown that the tumor immune microenvironment (TIME) plays a critical role in mediating antitumor immune response and immunotherapeutic response [19]. Thus, classification of MSI-L/MSS COADs based on the TIME may identify their subtypes responsive to immunotherapies. To this end, we aimed to identify subtypes of MSI-L/MSS COADs on the basis of the enrichment levels of 28 immune cells. We further analyzed molecular and clinicopathologic features of these subtypes, including pathway enrichment, genomic features, tumor phenotypes, and clinical outcomes. The identification of immune-specific subtypes may provide new insights into the pathogenesis of MSI-L/MSS COAD and potential clinical implications for immunotherapy of this disease.

## 2. **Materials and Methods**

### 2.1. Data Acquisition and Processing

We downloaded The Cancer Genome Atlas Colon Adenocarcinoma (TCGA-COAD) dataset, including RNA-Seq gene expression profiles (RSEM normalized), somatic mutation profiles (“maf” file), somatic copy number alterations (SCNAs) (“SNP6” files), protein expression profiles (Reverse Phase Protein Array (RPPA), normalized), pathological slides data, and clinical data, from the genomic data commons (GDC) data portal (https://portal.gdc.cancer.gov/). We obtained other COAD transcriptomic datasets (GSE39582, GSE41258, and GSE143985) from the NCBI gene expression omnibus (GEO) (https://www.ncbi.nlm.nih.gov/geo/). We also downloaded a single-cell RNA sequencing (scRNA-seq) dataset (GSE132465 [20]) for COAD from the NCBI GEO. A summary of these datasets is shown in Supplementary Table S1.

### 2.2. Single-Sample Gene Set Enrichment Analysis

Based on gene expression profiles, the single-sample gene set enrichment analysis (ssGSEA) [21] calculates the enrichment score of a gene set in a sample, which represents the degree to which the genes in the gene set are coordinately up- or downregulated in the sample. We used the ssGSEA to evaluate the enrichment of immune cells, biological processes, and pathways in tumors based on the expression profiles of their marker or pathway genes. The marker or pathway genes are presented in Supplementary Table S2. We performed the ssGSEA with the R package “GSVA.”

### 2.3. Clustering Analysis

We hierarchically clustered MSI-L/MSS COAD to uncover its immune subtypes based on the enrichment scores of 28 immune cell types. These cell types included CD56-bright natural killer (NK) cells, effector memory CD4 T cells, eosinophil, CD56-dim NK cells, type 17 T helper cells, activated B cells, monocytes, memory B cells, activated CD4 T cells, type 2 T helper cells, plasmacytoid dendritic cells, neutrophils, macrophages, effector memory CD8 T cells, myeloid-derived suppressor cell (MDSC), immature B cells, T follicular helper cells, NK cells, immature dendritic cells, mast cells, type 1 T helper cells, activated dendritic cells, central memory CD4 T cells, gamma delta T cells, central memory CD8 T cells, regulatory T cells, activated CD8 T cells, and natural killer T cells [22]. The enrichment score of an immune cell type in a tumor was the ssGSEA score of its marker gene set in the tumor. Before clustering, we normalized the ssGSEA scores by *z*-score and transformed them into distance matrices by the R function “dist” with the parameter method = “Euclidean.” We performed hierarchical clustering using the function “hclust” in the R package “Stats” with the parameters method = “ward.D2” and members = NULL.

### 2.4. Class Prediction

To predict the immune subtypes of MSI-L/MSS COAD by the immune cell types, we first normalized attribute values (ssGSEA scores of immune cell types) by *z*-score. We used the random forest (RF) algorithm to perform the class prediction. In the RF, the number of trees was set to 100, and the attributes included all 28 immune cell types. We reported the accuracy and weighted *F*-score as the prediction performance. We implemented the class prediction by Weka (version 3.8.5) [23].

### 2.5. Survival Analysis

We used the Kaplan-Meier (K-M) model [24] to compare overall survival (OS) and disease-free survival (DFS) time among different groups of cancer patients. K-M curves were used to display the survival time differences, and log-rank tests were utilized to evaluate the significance of survival time differences. We performed survival analyses in TCGA-COAD and GSE39582 in which related data were available.

### 2.6. Evaluation of TMB, SCNA, ITH, Immune Scores, and Tumor Purity in Tumors

TMB was defined as the total count of somatic mutations in the tumor. We used GISTIC2 [25] to calculate *G*-scores in tumors with the input of “SNP6” files. The *G*-score indicates the amplitude of the SCNA and the frequency of its occurrence across a group of samples [25]. We used the DITHER algorithm [26] to evaluate ITH levels, which scores ITH at the DNA level. We utilized ESTIMATE [27] to evaluate immune scores and tumor purity for bulk tumors. The immune score indicates the tumor immune infiltration level and tumor purity the proportion of tumor cells in a bulk tumor.

### 2.7. Pathway and Gene Ontology (GO) Analysis

To identify pathways highly enriched in one class versus another class, we first identified upregulated genes in the class relative to another class using Student's *t* test with a threshold of false discovery rate (FDR) < 0.05 and fold change (FC) > 2. By inputting the upregulated genes into the GSEA web tool [28], we obtained highly enriched KEGG [29] pathways with a threshold of FDR < 0.05. In addition, we used the weighted gene coexpression network analysis (WGCNA) [30] to identify the gene modules of coexpressed genes. Based on the expression correlations between the hub genes in gene modules, we identified the GO terms having significant correlations with specific traits. We performed the WGCNA analysis with the R package “WGCNA” (version 1.68).

### 2.8. scRNA-Seq Data Analysis

We analyzed a scRNA-seq dataset (GSE132465 [20]) for MSS COAD. The gene expression values have been normalized by natural log transformation of transcripts per million (TPM). We utilized the single-cell consensus clustering (SC3) method [31] to perform unsupervised clustering of cancer cells in each immune subtype. We used the inferCNV algorithm [32] to infer large-scale DNA copy number variations (CNVs) in cancer cells versus normal cells. We normalized the CNV values of cells output by inferCNV by subtracting the average of the maximum and minimum values in the matrix of CNV values to make the “0” representing the copy number in normal cells. We defined the CNV score of each cell as the average of quadratic sum of the CNV values for all genes.

### 2.9. Statistical Analysis

We used Student's *t* test (two-tailed) to compare two classes of normally distributed data, including gene expression levels, protein expression levels, and the ratios of two different immune signatures. The ratios were the log2-transformed values of the average expression levels of all marker genes in an immune signature divided by those of all marker genes in another immune signature. In comparisons of two classes of nonnormally distributed data, such as ssGSEA scores of gene sets, TMB, ITH, immune scores, and tumor purity, we used the Mann–Whitney *U* test (one-tailed). We utilized the Spearman method to evaluate the correlation between pathway activities (ssGSEA scores) and immune scores. The Fisher's exact test was used to analyze contingency tables. To adjust for *P* values in multiple tests, we calculated FDR with the Benjamini and Hochberg method [33]. We performed all statistical analyses with the R programming language (version 3.6.0).

## 3. Results

### 3.1. Clustering Analysis Identifies Three Immune Subtypes of MSI-L/MSS COAD

**Figure 1:**
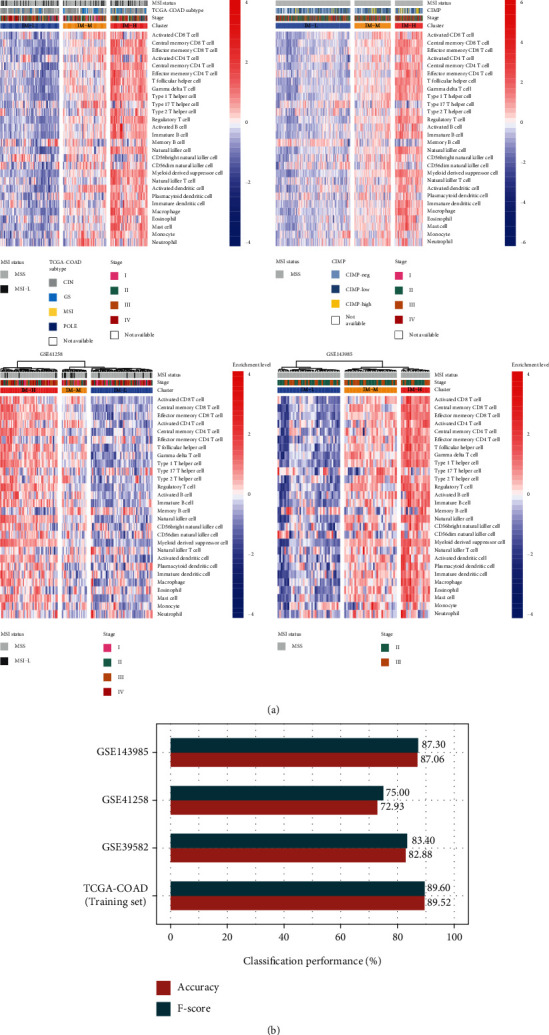
Hierarchical clustering of MSI-L/MSS COAD bulk tumors based on the enrichment of 28 immune cell types. (a) Clustering analyses uncovering three immune subtypes of MSI-L/MSS COAD, IM-H, IM-M, and IM-L, which have high, medium, and low immune cell enrichment scores, respectively, consistently in four datasets. (b) Prediction of the three immune subtypes of MSI-L/MSS COAD by random forest based on the enrichment scores of 28 immune cell types. TCGA-COAD dataset as the training set and the other three datasets as test sets. The 10-fold cross-validation results in the training set and prediction results in the other datasets are shown.

Based on the enrichment scores of 28 immune cell types, we identified three subtypes of MSI-L/MSS COAD by hierarchical clustering, consistently in the four bulk transcriptome datasets (TCGA-COAD, GSE39582, GSE41258, and GSE143985) (Figure 1). The three subtypes had high, medium, and low enrichment scores of the immune cells, termed IM-H, IM-M, and IM-L, respectively. The consistent clustering results demonstrate the reproducibility of this classification method. Furthermore, to explore whether this classification is predictable, we used the TCGA-COAD dataset as the training set and the other three datasets as test sets. The 10-fold cross-validation (CV) accuracy in the training set was 89.52%. The prediction accuracies were 82.88%, 72.93%, and 87.06% in GSE39582, GSE41258, and GSE143985, respectively (Figure 1(b)). The weighted *F*-scores in these predictions were 89.60%, 83.40%, 75.00%, and 87.30% for TCGA-COAD, GSE39582, GSE41258, and GSE143985, respectively (Figure 1(b)). Overall, these results demonstrate that the immunological classification of MSI-L/MSS COAD is reproducible and predictable.

**Figure 2:**
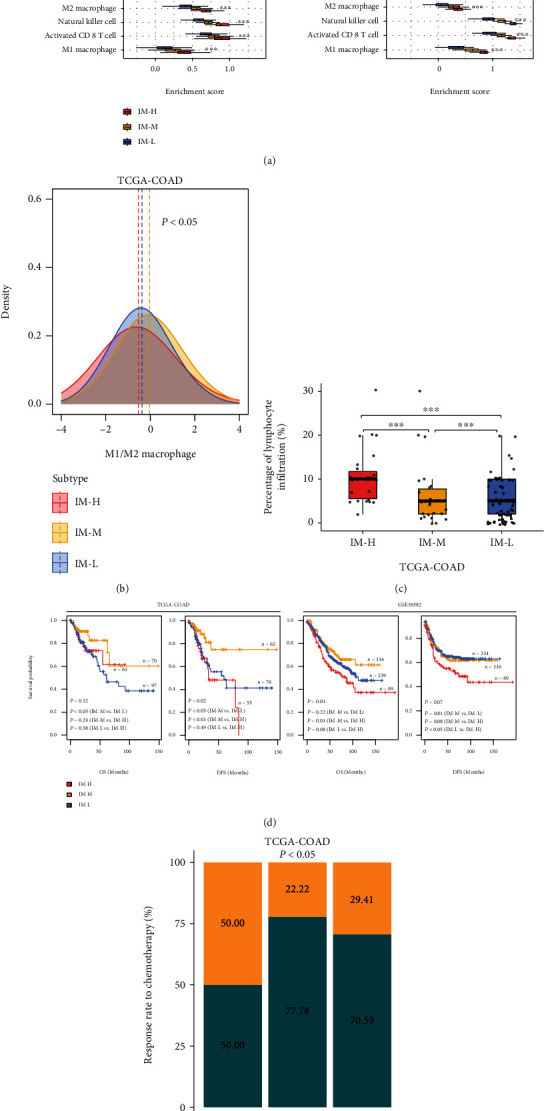
Comparisons of immune signature enrichment and clinical outcomes among the three immune subtypes. Comparisons of the enrichment scores of immunostimulatory signatures (M1 macrophages, activated CD8 T cells, and NK cells) and immunosuppressive signatures (M2 macrophages, regulatory T cells, myeloid-derived suppressor cells (MDSCs), and *PD-L1*) (a), ratios of immunostimulatory to immunosuppressive signatures (M1/M2 macrophages) (b), and the percentage of tumor-infiltrating lymphocytes (TILs) (c) among the three immune subtypes. The Kruskal–Wallis test (a), one-way ANOVA (b), and one-tailed Mann–Whitney *U* test (c). *P* values are shown. ∗*P* < 0.05, ∗∗*P* < 0.01, ∗∗∗*P* < 0.001, and ^ns^*P* ≥ 0.05. It also applies to the following figures. (d) Comparisons of overall survival (OS) and disease-free survival (DFS) rates among the immune subtypes by the Kaplan–Meier curves. The log-rank test *P* values are shown. (e) Comparisons of the response (complete or partial response) rates to chemotherapy among the three immune subtypes in TCGA-COAD. The chi-square test *P* value is shown.

Notably, both immunostimulatory signatures (such as M1 macrophages, activated CD8 T cells, and NK cells) and immunosuppressive signatures (such as M2 macrophages, regulatory T cells, MDSCs, and *PD-L1*) showed the highest enrichment scores in IM-H and the lowest enrichment scores in IM-L (one-tailed Mann–Whitney *U* test or two-tailed Student's *t* test, *P* < 0.001) (Figure 2(a)). However, the ratios of immunostimulatory to immunosuppressive signatures (M1/M2 macrophages) were the highest in IM-M among the three subtypes (two-tailed Student's *t* test, *P* < 0.05) in TCGA-COAD (Figure 2(b)). We further compared the percentages of tumor-infiltrating lymphocytes (TILs) among the three subtypes provided by the pathology slide data in TCGA-COAD. As expected, the percentages of TILs were significantly higher in IM-H than in IM-M and IM-L (*P* < 0.001) (Figure 2(c)). Taken together, these results confirmed that IM-H and IM-L had the highest and lowest enrichment of immune signatures, respectively.

We compared OS and DFS rates among the immune subtypes of MSI-L/MSS COAD in TCGA-COAD and GSE39582, in which related data were available. Survival analyses showed that IM-M had better DFS than IM-H and IM-L (log-rank test, *P* < 0.05) in TCGA-COAD, while there was no significant difference of DFS between IM-H and IM-L in this cohort (*P* = 0.49) (Figure 2(d)). Moreover, IM-M displayed better OS than IM-L in TCGA-COAD (*P* < 0.05) (Figure 2(d)). In GSE39582, IM-M showed better OS than IM-H (*P* = 0.01), and IM-L had better DFS than IM-H (*P* < 0.05) (Figure 2(d)). Taken together, these results indicate that IM-M and IM-H likely have the best and worst survival, respectively. In addition, we compared the response rate to chemotherapy among the three immune subtypes in TCGA-COAD. We found that the response (complete or partial response) rate to chemotherapy followed the pattern IM-M (77.78%) > IM-L (70.59%) > IM-H (50.00%) (Figure 2(e)), supporting the results of prognostic analysis.

### 3.2. The Immune Subtypes of MSI-L/MSS COAD Have Significantly Different Phenotypic and Molecular Features

**Figure 3:**
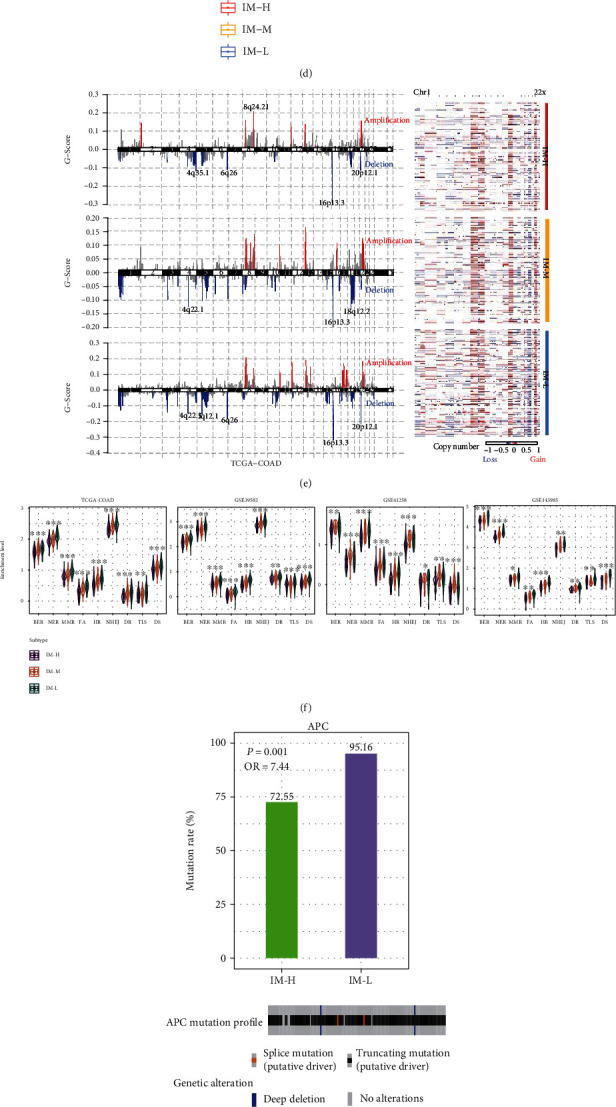
Comparisons of phenotypic and molecular features among the immune subtypes. Comparisons of the tumor progressive phenotypic or molecular features (a), oncogenic pathways (b), tumor purity (c), intratumor heterogeneity (ITH) (d), copy number alteration (G-scores) (e), and DNA damage repair (DDR) pathways (f) among the three immune subtypes. The Kruskal–Wallis test (a and b) and one-tailed Mann–Whitney *U* test (c, d, and f) *P* values are shown. (g) Comparisons of *APC* mutation rate between the high (IM-H) and low (IM-L) immune subtypes and showing *APC* mutation profiles in IM-L. (h) Comparisons of *NOTCH3* mutation rate between the medium (IM-M) and the other two (IM-H/L) immune subtypes and overall survival rate between *NOTCH3*-mutated and *NOTCH3*-wild-type MSI-L/MSS COAD in TCGA-COAD. The Fisher's exact test *P* values are shown in (g and h). (i) Heatmap showing the proteins with significant expression differences between IM-H and IM-L in TCGA-COAD (two-tailed Student's *t* test, FDR < 0.05).

We observed that the phenotypic or molecular features indicative of tumor progression, such as stemness, epithelial-mesenchymal transition (EMT), proliferation, invasion, and metastasis, were significantly more enriched in IM-H compared to IM-M and IM-L (*P* < 0.05) (Figure 3(a)). Furthermore, numerous oncogenic pathways displayed significantly higher enrichment in IM-H versus IM-M and IM-L (*P* < 0.001), including the pathways of PI3K-Akt, VEGF, JAK-STAT, RAS, HIF-1, and MAPK signaling (Figure 3(b)). In contrast, tumor purity was significantly lower in IM-H than in IM-M and IM-L (*P* < 0.001) (Figure 3(c)); ITH scores followed the pattern IM-H < IM-M < IM-L (*P* < 0.05) (Figure 3(d)).

There was no significant difference of TMB among the three immune subtypes of MSI-L/MSS COAD (Kruskal–Wallis test, *P* = 0.568). However, tumor aneuploidy, namely, copy number alteration (CNA), showed significant difference among the subtypes, as evidenced by that the *G*-scores of copy number amplifications and deletions were the highest in IM-L and the lowest in IM-H (Figure 3(e)). Since the *G*-score represents the amplitude of CNA and the frequency of its occurrence across a group of samples [25], it indicated that IM-L and IM-H had the highest and lowest CNA, respectively. This result is in agreement with the previous studies showing that tumor aneuploidy correlates with reduced antitumor immune response [34]. Furthermore, we compared the enrichment scores of nine major DNA damage repair (DDR) pathways among the subtypes. These pathways included mismatch repair, base excision repair, nucleotide excision repair, the Fanconi anemia (FA) pathway, homology-dependent recombination, nonhomologous DNA end joining, direct damage reversal/repair, translesion DNA synthesis, and damage sensor [35]. Notably, the enrichment scores of nine DDR pathways followed the pattern IM-L > IM-M > IM-H (*P* < 0.05) (Figure 3(f)). Together, these results indicated that IM-L and IM-H had the highest and lowest genomic instability, respectively.

We found 14 genes more frequently mutated in IM-H than in IM-L (Fisher's exact test, *P* < 0.05; odds ratio (OR) > 3). These genes included *USH2A*, *HMCN1*, *PTPRT*, *ADAMTSL3*, *TDRD6*, *TRO*, *TCHH*, *ATP8A2*, *CCDC9*, *DCDC5*, *FADS3*, *LRRC7*, *NOTCH3*, and *SPG20*. Notably, the mutations in each of these genes were correlated with significantly higher immune scores in MSI-L/MSS COAD (*P* < 0.05) (Supplementary Table S3). On the contrary, seven genes showed a significantly higher mutation rate in IM-L than in IM-H (*P* < 0.04; OR > 7), including *APC*, *CHD5*, *DCLK1*, *FBXL7*, *COL6A6*, *KRTAP10-10*, and *PCDHGA5*. *APC* is a tumor suppressor gene involved in the regulation of WNT signaling, whose mutations are prevalent in nonhypermutated tumors [36]. The *APC* mutations in IM-L were mainly truncating mutations (Figure 3(g)), which may initiate chromosome instability [37, 38]. This could partially explain why IM-L had higher genomic instability than IM-H. Furthermore, we compared gene mutation profiles between IM-M and IM-H/L. Notably, IM-H/L displayed a significantly higher frequency of *CUBN* mutations than IM-M (*P* = 0.037; OR = 7.15). A previous study has demonstrated that *CUBN* mutations might promote the malignancy of CRC [39]. There were 28 genes showing a significantly higher mutation rate in IM-M than in IM-H/L (*P* < 0.05; OR > 2). Noticeably, the mutation frequency of *NOTCH3* was significantly higher in IM-M than in IM-H/L (*P* = 0.011; OR = 5.16), and its mutation was associated with a higher OS rate in MSI-L/MSS COAD (*P* = 0.033) (Figure 3(h)).

We compared the expression of 226 proteins among the subtypes. We found 45 proteins significantly upregulated in IM-H relative to IM-L (two-tailed Student's *t* test, FDR < 0.05) (Figure 3(i) and Supplementary Table S4). Many of these proteins were protein kinases involved in signal transduction, such as p38_MAPK, MEK1, MAPK_pT202_Y204, and Lck. Several cluster of differentiation CD molecules were also in the list of the 45 proteins, such as CD20, CD26, and CD31, supporting the higher tumor immunity in IM-H versus IM-L. The 45 proteins also included some molecules involved in immune regulation, such as ETS1 [40], Annexin-1 [41, 42], and Lck [43]. In contrast, 48 proteins showed significantly higher expression levels in IM-L than in IM-H (Figure 3(i) and Supplementary Table S4). Notably, two DNA mismatch repair proteins (MSH2 and MSH6) were in the list of the 48 proteins. In addition, several tumor suppressors, such as Rb, tuberin, and E-cadherin, were upregulated in IM-L relative to IM-H. The higher enrichment of these tumor suppressors in IM-L could explain why IM-L had a better relapse-free survival rate than IM-H.

### 3.3. Identification of Pathways and GO Highly Enriched in the Immune Subtypes of MSI-L/MSS COAD

**Figure 4:**
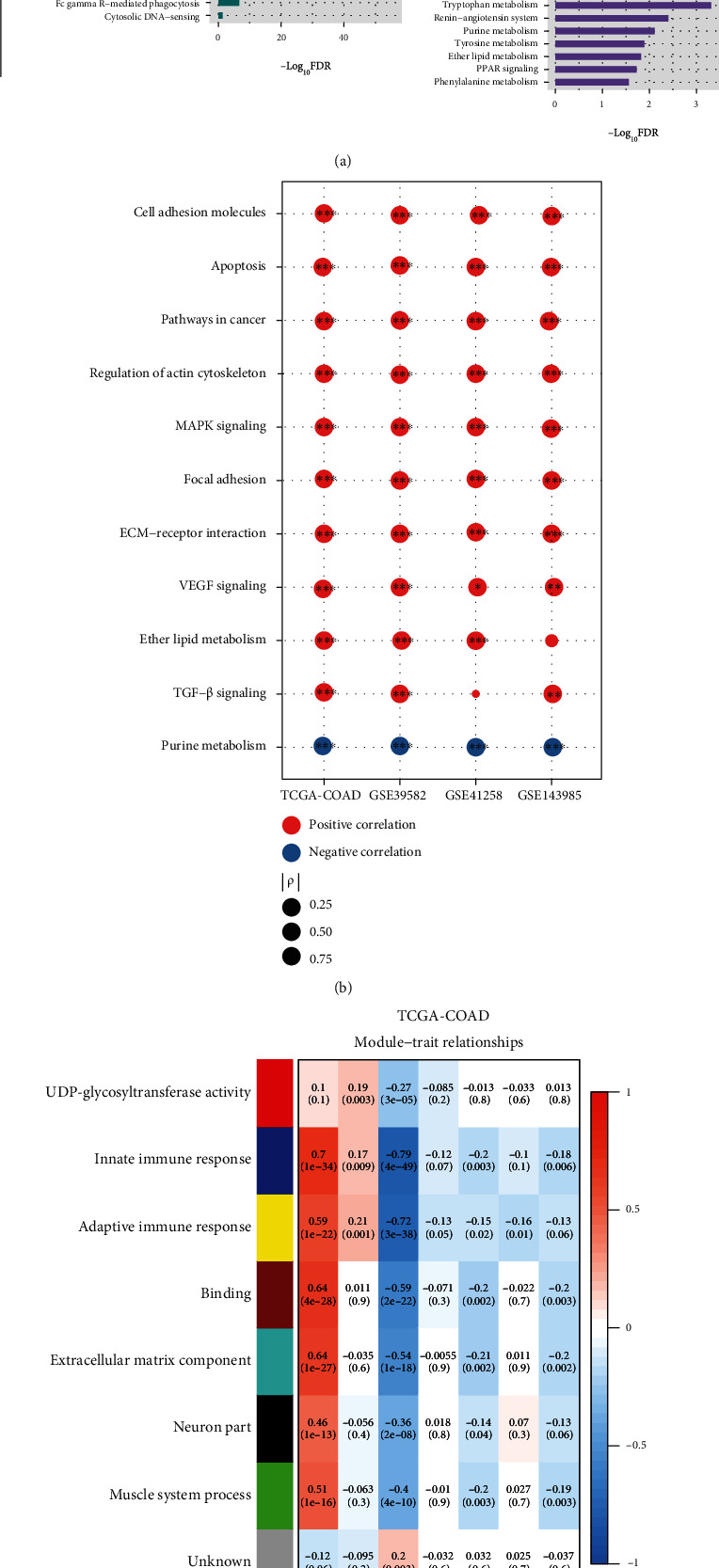
Pathways and Gene Ontology (GO) enriched in the immune subtypes. (a) The KEGG pathways upregulated in IM-H versus IM-L in TCGA-COAD. (b) Spearman's correlations between the enrichment scores of pathways upregulated in IM-H and immune scores in MSI-L/MSS COAD. The correlation coefficients (*ρ*) and *P* values are shown. (c) The gene modules and their representative GO terms significantly differentiating MSI-L/MSS COAD by the immune subtypes and survival prognosis in TCGA-COAD.

Pathway analysis by GSEA [28] identified numerous KEGG pathways highly enriched in IM-H versus IM-L in TCGA-COAD. These pathways were mainly involved in immune, stromal, oncogenic, and metabolic signatures (Figure 4(a)). The immune-related pathways included cytokine-cytokine receptor interaction, hematopoietic cell lineage, chemokine signaling, intestinal immune network for IgA production, leukocyte transendothelial migration, complement and coagulation cascades, primary immunodeficiency, Toll-like receptor signaling, T cell receptor signaling, natural killer cell mediated cytotoxicity, B cell receptor signaling, Jak-STAT signaling pathway, NOD-like receptor signaling, Fc epsilon RI signaling, antigen processing and presentation, Fc gamma R-mediated phagocytosis, and cytosolic DNA-sensing pathway. It confirmed that IM-H had higher immune activity than IM-L. The stromal signature-related pathways included cell adhesion molecules, ECM-receptor interaction, focal adhesion, regulation of actin cytoskeleton, and tight junction. The cancer-related pathways included pathways in cancer, MAPK, TGF-*β*, VEGF, and Hedgehog signaling. The metabolism-related pathways included tryptophan metabolism, renin-angiotensin system, purine metabolism, tyrosine metabolism, ether lipid metabolism, PPAR signaling, and phenylalanine metabolism. As expected, in addition to the immune-related pathways, most of the other pathways showed significantly positive correlations of their enrichment scores with immune scores in MSI-L/MSS COAD (Spearman's correlation, *P* < 0.05) (Figure 4(b)).

WGCNA [30] identified seven gene modules significantly differentiating MSI-L/MSS COAD by the subtypes and survival prognosis in TCGA-COAD (Figure 4(c)). Notably, six gene modules (highlighted in blue, yellow, brown, turquoise, black, and green, respectively) were significantly upregulated in IM-H, while they were downregulated in IM-L (*P* < 0.001). Interestingly, these gene modules' enrichment was consistently and negatively correlated with OS and/or DFS time (*P* < 0.05) (Figure 4(c)). The representative GO terms for these gene modules included innate immune response, adaptive immune response, binding, extracellular matrix, neuron part, and muscle system process (Figure 4(c)). It is in agreement with the previous results that immune and stromal pathways are upregulated in IM-H relative to IM-L. Besides, there was a gene module (highlighted in red) significantly upregulated in IM-M but downregulated in IM-L (*P* < 0.01). The representative GO term for this gene module was UDP-glycosyltransferase activity. UDP-glycosyltransferase activity accelerates metabolic inactivation of drug therapies to produce drug resistance and affects cancer progression [44, 45]. That is, patients in the IM-L subtype are more likely to benefit from drug treatment because of low drug resistance.

### 3.4. Clustering Analysis Identifies Three Immune Subtypes of MSI-L/MSS COAD Single Cells

**Figure 5:**
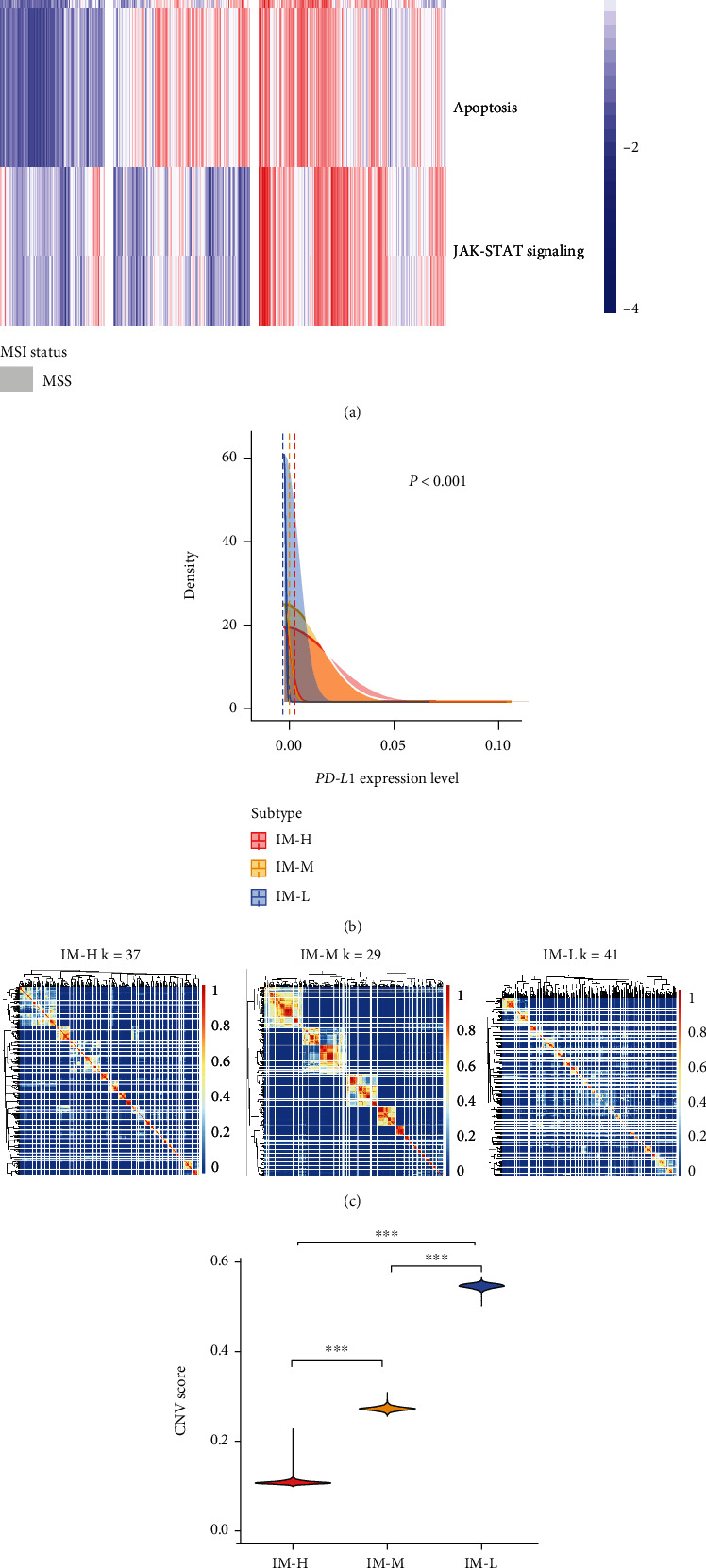
Validation of the immune signature enrichment-based subtyping method in MSS COAD single cells. (a) Hierarchical clustering of 12484 cancer cells from 16 MSS COAD patients based on the enrichment scores of four immune-related pathways identifying three subtypes. (b) Comparisons of *PD-L1* expression levels among the subtypes of cancer cells. The one-way ANOVA test *P* value is shown. (c) Unsupervised clustering of each subtype of single cells by SC3 [31] identifying 37, 29, and 41 clusters in IM-H, IM-M, and IM-L, respectively. (d) Comparisons of the inferred copy number variations (CNVs) by inferCNV [32] among the three immune subtypes of single cells. The one-tailed Mann–Whitney *U* test *P* values are shown. (e) Comparisons of the enrichment of T cell subpopulations among the immune subtypes. The two-tailed Student's *t* test *P* values are shown.

We performed a similar clustering analysis of MSI-L/MSS COAD single cells using a scRNA-seq dataset (GSE132465). This dataset involved gene expression profiles in 12,484 cancer cells from 16 MSS COAD patients. We hierarchically clustered these cancer cells based on the enrichment scores of four immune-related pathways, including antigen processing and presentation, apoptosis, JAK-STAT signaling, and PD-L1 expression pathway in cancer. We used these pathways instead of the previous 28 immune cell types in clustering single cells because these pathways are likely expressed in cancer cells themselves. Likewise, we identified three clusters of these cancer single cells (IM-H, IM-M, and IM-L), which had high, medium, and low enrichment scores of these pathways (Figure 5(a)). As expected, *PD-L1* expression levels were the highest in IM-H and the lowest in IM-L (*P* < 0.001) (Figure 5(b)). We further performed unsupervised clustering of each subtype of these single cells by SC3 [31] and identified 37, 29, and 41 cell clusters in IM-H, IM-M, and IM-L, respectively (Figure 5(c)). It indicated that IM-L and IM-M had the highest and lowest heterogeneity of cancer cells. Furthermore, we observed that the inferred CNVs by inferCNV [32] followed the pattern IM-L > IM-M > IM-H (*P* < 0.001) (Figure 5(d)). These results were consistent with those obtained in bulk tumors, supporting that IM-L and IM-H had the highest and lowest genomic instability, respectively, at the single-cell level. Based on the cell clustering results, we calculated the proportions of cancer cells of each patient in each subtype of IM-H, IM-M, and IM-L and assigned each patient into the subtype which involved the highest proportion of cancer cells of that patient. We further compared the enrichment levels of several T cell subpopulations among IM-H, IM-M, and IM-L patients, including CD4+ FOXP3 for regulatory CD4+ T cells, CD4+ IL7R for resting CD4+ T cells, CD4+ CXCL13 for activated CD4+ T cells, and CD8+ GZMB T cells. The enrichment levels of these T cell subpopulations were the average expression levels of their marker genes (Supplementary Table S2). Interestingly, the CD4+ FOXP3 T cell enrichment was the highest in IM-H and the lowest in IM-M (*P* < 0.05) (Figure 5(e)). However, the CD4+ CXCL13 T cell enrichment followed an opposite pattern: IM-H < IM-L < IM-M (*P* < 0.001). In addition, the CD4+ IL7R T cell enrichment was the highest in IM-L and the lowest in IM-M (*P* < 0.001), while the CD8+ GZMB T cell enrichment followed an opposite pattern: IM-L < IM-H < IM-M (*P* <0.001). These results indicated that immunostimulatory signatures were the most enriched in IM-M, while immunosuppressive signatures were the least enriched in this subtype. It is consistent with the finding of the highest ratios of immunostimulatory to immunosuppressive signatures in IM-M among the three subtypes in bulk tumors.

## 4. Discussion

**Figure 6:**
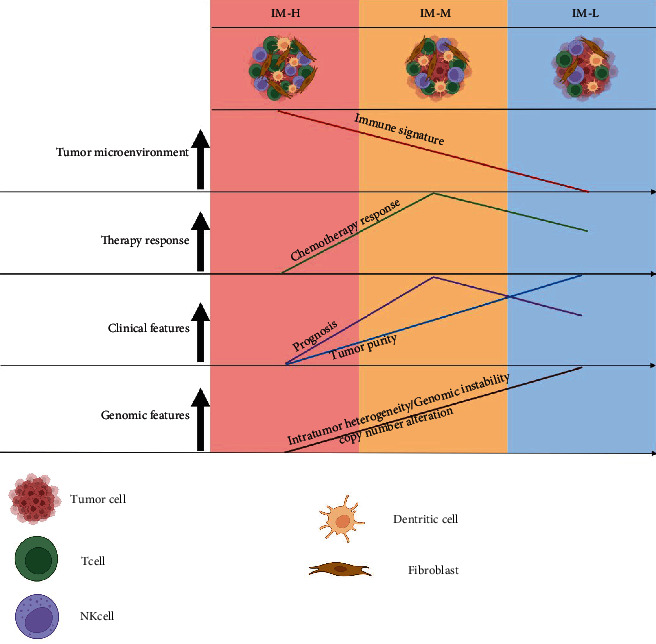
A summary of molecular and clinical characteristics of the three immune subtypes. The figure was created with http://BioRender.com.

Although MSI has been identified as an indicator for antitumor immune response and immunotherapy response, only 15% of COAD patients are endowed with this feature. This study focused on MSI-L/MSS COAD and identified its immune subtypes based on the immune features displayed in the TIME. We identified three immune subtypes of MSI-L/MSS COAD (IM-H, IM-M, and IM-L), which had high, medium, and low immune signature scores, respectively. We demonstrated that this subtyping method was reproducible and predictable by analyzing five different datasets, including four bulk tumor datasets and one single cell dataset. IM-H was characterized by high immunity, high stemness, strong potential of proliferation, invasion and metastasis, EMT, high expression of oncogenic pathways, low tumor purity, low ITH, genomic instability, inferior response to chemotherapy, and unfavorable survival prognosis (Figure 6). IM-M was characterized by the highest ratio of immunostimulatory to immunosuppressive signatures, the best response to chemotherapy as well as survival prognosis. IM-L was characterized by low immunity, high tumor purity, high ITH, and genomic stability. It is interesting to observe that IM-H has the worst survival among these subtypes, although this subtype displays the “hottest” TIME. The inverse association between tumor immune infiltration levels and clinical outcomes has also been observed in some other cancer types, such as glioma [46] and prostate cancer [47]. The main reason for the inverse association between tumor immune infiltration levels and clinical outcomes could be that the strong immune infiltration leads to tumor progression-promoting inflammation [48]. Our data indicate that this inflammation is in fact antitumor immunosuppression as IM-H displays the highest expression of various immunosuppressive signatures, including M2 macrophages, regulatory T cells, MDSCs, and *PD-L1*. Another interesting finding is that IM-M instead of IM-L has the best survival prognosis. A possible explanation for the best prognosis in IM-M could be that the immune-mediated tumor killing is the strongest in this subtype, as evidenced by the highest ratio of immunostimulatory to immunosuppressive signatures in bulk tumors, as well as the highest enrichment of immunostimulatory signatures (such as activated CD4+ T cells and CD8+ GZMB T cells) and the lowest enrichment of immunosuppressive signatures (such as regulatory CD4+ T cells and resting CD4+ T cells) in single cells in IM-M. In addition, previous studies [49, 50] have demonstrated that relative proportions of M1 macrophages and M2 macrophages correlates positively with survival prognosis in COAD. It is in line with the highest ratio of M1/M2 macrophages in IM-M. Nevertheless, by contrast, the association between tumor immune infiltration levels and clinical outcomes is positive in many other cancer types, such as gastric cancer [51], head and neck squamous cell cancer [52], and triple-negative breast cancer [53]. Hence, the association between the TIME and malignancy is complex and cancer type dependent.

Among the three subtypes of COAD defined by TCGA (MSI, GS, and CIN) [54], MSI-L/MSS constituted around 82% of CIN. Notably, IM-L harbored the highest proportion of CIN cases (47.93% in IM-L versus 25.44% in IM-H and 26.63% in IM-M) (Fisher's exact test, *P* < 0.05). CIN is characterized by marked aneuploidy that is consistent with the highest CNA exhibited in IM-L. Furthermore, it conforms to the previous findings that aneuploidy correlates with reduced antitumor immune response [34]. Another previous study [55] identified four consensus subtypes of CRC (CMS1, CMS2, CMS3, and CMS4), by integrating six different classification systems. We found that 71.43% of IM-H cases were included in CMS4, compared to 23.81% of IM-M and 4.76% of IM-L in CMS4 (Fisher's exact test, *P* < 0.001). In fact, there were common features between CMS4 and IM-H, including EMT upregulation, TGF-*β* signaling pathway activation, stromal invasion, and worse prognosis. Meanwhile, 66.23% of IM-L cases were involved in CMS2, compared to 2.60% of IM-H and 31.17% of IM-M in CMS2. Again, CMS2 shared several prominent characteristics with IM-L, including high CIN, low immunogenicity, and decreased relapse rates. A previous study [56] molecularly classified CRC based on the expression levels of EMT-associated markers and identified three subtypes: epithelial, mesenchymal, and hybrid. Among the MSI-L/MSS COAD immune subtypes we identified, IM-H should have the highest overlaps with the mesenchymal subtype for its highest EMT scores, while IM-L should have the highest overlaps with the epithelial subtype for its lowest EMT scores; IM-M likely has the highest overlaps with the hybrid subtypes. That study [56] indicated that the EMT-based classification of CRCs could identify the most aggressive subtype showing a mesenchymal phenotype, consistent with our results showing that IM-H has the worst clinical outcomes among the three immune subtypes of MSI-L/MSS COAD.

MSI-H is an established indicator for immunotherapy response for its high TMB, *PD-L1* expression, and TIL level. However, we found that IM-H COADs likely had significantly higher TIL levels than MSI-H COADs (*P* < 0.05). Moreover, *PD-L1* expression levels showed no significant difference between MSI-H and IM-H COADs in two of the three datasets (*P* > 0.35). These data indicate that a proportion of non-MSI-H tumors could also be propitious to immunotherapy. Thus, the immune signature enrichment-based subtyping of MSI-L/MSS COAD may identify non-MSI-H patients responding well to immunotherapy. In fact, the immunotherapy of MSI-L/MSS COAD has been under investigation by clinical trials [57]. In addition, the combination of immunotherapy with other therapies could be a promising direction in treating MSI-L/MSS COADs.

This study has several limitations. First, the results presented in this study were obtained by bioinformatics analyses but lack experimental validation. Our next step is to validate the results by in vitro and in vivo experiments. Second, although our classification has potential value in stratifying COAD patients responsive to immunotherapies, it needs to be verified with clinical data with immunotherapy information. It is also an objective of our future research.

## 5. Conclusions

Based on the enrichment scores of immune signatures, MSI-L/MSS COAD can be classified into three subtypes with high, medium, and low enrichment of immune signatures in the TIME. The immune-specific subtypes have significantly different TIME, tumor purity, stemness, tumor progression phenotypes, ITH, genomic features, response to chemotherapy, and survival prognosis. This study may provide new insights into COAD immunity, as well as identify non-MSI patients responsive to immunotherapy.

## Data Availability

The data used to support the findings of this study were presented in Supplementary Tables and from public databases: the genomic data commons (GDC) data portal (https://portal.gdc.cancer.gov/) and the NCBI gene expression omnibus (GEO) (https://www.ncbi.nlm.nih.gov/geo/).

## Supplementary Materials

Supplementary Materials Table S1: a summary of the datasets analyzed. Table S2: the gene sets representing immune cells, pathways, and biological processes. Table S3: genes with significantly different mutation rates between the immune subtypes of MSI-L/MSS COAD in TCGA-COAD. Table S4: 93 proteins differentially expressed between IM-H and IM-L in TCGA-COAD.
