# Supplementary material for: Dissection of Immune Profiles in Microsatellite Stable and Low Microsatellite Instability Colon Adenocarcinoma by Multiomics Data Analysis
Source: J Oncol. 2022 Apr 15;2022:8588164. doi: 10.1155/2022/8588164 (PMC9033404; doi:10.1155/2022/8588164)
Supplement: Supplementary Materials — Table S1: a summary of the datasets analyzed. Table S2: the gene sets representing immune cells, pathways, and biological processes. Table S3: genes with significantly different mutation rates between the immune subtypes of MSI-L/MSS COAD in TCGA-COAD. Table S4: 93 proteins differentially expressed between IM-H and IM-L in TCGA-COAD. [file 8588164.f1.docx]

| **Table S1. A summary of the datasets analyzed** | | |
| --- | --- | --- |
| **Dataset** | **Number of samples** | **Source** |
| TCGA-COAD | 229 (bulk tumors) | https://portal.gdc.cancer.gov/projects/TCGA-COAD |
| GSE39582 | 444 (bulk tumors) | https://www.ncbi.nlm.nih.gov/geo/query/acc.cgi?acc=GSE39582 |
| GSE41258 | 133 (bulk tumors) | https://www.ncbi.nlm.nih.gov/geo/query/acc.cgi?acc=GSE41258 |
| GSE143985 | 85 (bulk tumors) | https://www.ncbi.nlm.nih.gov/geo/query/acc.cgi?acc=GSE143985 |
| GSE132465 | 12484 (cancer cells), 20529 (non-cancer cells) | https://www.ncbi.nlm.nih.gov/geo/query/acc.cgi?acc=GSE132465 |

| **Table S2. The gene sets representing immune cells, pathways, and biological processes** | | |
| --- | --- | --- |
| **28 immune cell types for clustering bulk tumors** | Activated CD8 T cell | *ADRM1, AHSA1, C1GALT1C1, CCT6B, CD37, CD3D, CD3E, CD3G, CD69, CD8A, CETN3, CSE1L, GEMIN6, GNLY, GPT2, GZMA, GZMH, GZMK, IL2RB, LCK, MPZL1, NKG7, PIK3IP1, PTRH2, TIMM13, ZAP70* |
|  | Central memory CD8 T cell | *ACTN4, ADAM12, ADCY9, F13A1, FCER1G, FCGR3B, FGF7, FKBP4, GLUD1, GM2A, GUSB, IL1RN, NOL11, NTRK1, RARA, RNF128, SIGLEC1, TNFRSF11A, TOX4, UBA52, ULBP1* |
|  | Effector memory CD8 T cell | *ACAP1, APOL3, ARHGAP10, ATP10D, C3AR1, CCR5, CD160, CD55, CFLAR, CMKLR1, DAPP1, FCRL6, FLT3LG, GZMM, HAPLN3, HLA-DMB, HLA-DPA1, HLA-DPB1, IFI16, LIME1, LTK, NFKBIA, SETD7, SIK1, TRIB2* |
|  | Activated CD4 T cell | *AIM2, BIRC3, BRIP1, CCL20, CCL4, CCL5, CCNB1, CCR7, DUSP2, ESCO2, ETS1, EXO1, EXOC6, IARS, ITK, KIF11, KNTC1, NUF2, PRC1, PSAT1, RGS1, RTKN2, SAMSN1, SELL, TRAT1* |
|  | Central memory CD4 T cell | *ABHD3, AHNAK, ANXA2P2, AQP3, ATHL1, BMI1, BZW2, CD63, COL4A1, CYLD, ELMO2, FYN, GLIPR1, GSS, IFITM2, ITGB1, ITGB2, KLF5, LSP1, NDUFB9, PKM2, SFXN3, SIRPG, SMAD4, STX4, TRADD* |
|  | Effector memory CD4 T cell | *ATM, CASP3, CASQ1, CD300E, DARS, DOCK9, EXOSC9, EZH2, GDE1, IL34, NCOA4, NEFL, PDGFRL, PTGS1, REPS1, SCG2, SDPR, SIGLEC14, SIGLEC6, TAL1, TFEC, TIPIN, TPK1, UQCRB, USP9Y, WIPF1* |
|  | T follicular helper cell | *B3GAT1, CDK5R1, PDCD1, BCL6, CD200, CD83, CD84, FGF2, GPR18, CEBPA, CECR1, CLEC10A, CLEC4A, CSF1R, CTSS, DMN, DPP4, LRRC32, MC5R, MICA, NCAM1, NCR2, NRP1, PDCD1LG2, PDCD6, PRDX1* |
|  | Gamma delta T cell | *ACP5, AQP9, BTN3A2, C1orf54, CARD8, CCL18, CD209, CD33, CD36, CDK5, IL10RB, KLRF1, LGALS1, MAPK7, KLHL7, KRT80, LAMC1, LCORL, LMNB1, MEIS3P1, MPL, FABP1, FABP5, FADD, MFAP3L, MINPP1* |
|  | Type 1 T helper cell | *CD70, TBX21, ADAM8, AHCYL2, ALCAM, B3GALNT1, BBS12, BST1, CD151, CD47, CD48, CD52, CD53, CD59, CD6, CD68, CD7, CD96, CFHR3, CHRM3, CLEC7A, COL23A1, COL4A4, COL5A3, DAB1, DLEU7* |
|  | Type 17 T helper cell | *IL17A, IL17RA, C2CD4A, C2CD4B, CA2, CCDC65, CEACAM3, IL17C, IL17F, IL17RC, IL17RE, IL23A, ILDR1, LONRF3, SH2D6, TNIP2, ABCA1, ABCB1, ADAMTS12, ANK1, ANKRD22, B3GALT2, CAMTA1, CCR9, CD40, GPR44* |
|  | Type 2 T helper cell | *ASB2, CSRP2, DAPK1, DLC1, DNAJC12, DUSP6, GNAI1, LAMP3, NRP2, OSBPL1A, PDE4B, PHLDA1, PLA2G4A, RAB27B, RBMS3, RNF125, TMPRSS3, GATA3, BIRC5, CDC25C, CDC7, CENPF, CXCR6, DHFR, EVI5, GSTA4* |
|  | Regulatory T cell | *CCL3L1, CD72, CLEC5A, FOXP3, ITGA4, L1CAM, LIPA, LRP1, LRRC42, MARCO, MMP12, MNDA, MRC1, MS4A6A, PELO, PLEK, PRSS23, PTGIR, ST8SIA4, STAB1* |
|  | Activated B cell | *ADAM28, CD180, CD79B, BLK, CD19, MS4A1, TNFRSF17, IGHM, GNG7, MICAL3, SPIB, HLA-DOB, IGKC, PNOC, FCRL2, BACH2, CR2, TCL1A, AKNA, ARHGAP25, CCL21, CD27, CD38, CLEC17A, CLEC9A, CLECL1* |
|  | Immature B cell | *CD22, CYBB, FAM129C, FCRL1, FCRL3, FCRL5, FCRLA, HDAC9, HLA-DQA1, HVCN1, KIAA0226, NCF1, NCF1B, P2RY10, SP100, TXNIP, STAP1, TAGAP, ZCCHC2* |
|  | Memory B cell | *AICDA, CCNA2, CDKN3, CLCN5, ENPP1, FCER1A, FCRL4, MYC, RUNX2, SORL1, SOX5, STAT5A, STAT5B, TLR9* |
|  | Natural killer cell | *AKT3, AXL, BST2, CDH2, CRTAM, CSF2RA, CTSZ, CXCL1, CYTH1, DAXX, DGKH, DLL4, DPYD, ERBB3, F11R, FAM27A, FAM49A, FASLG, FCGR1A, FN1, FSTL1, FUCA1, GBP3, GLS2, GRB2, LST1* |
|  | CD56bright natural killer cell | *ABAT, C11orf75, C5orf15, CDHR1, DCAF12, DYNLL1, GPR137B, HCP5, HDGFRP2, KRT86, MLST8, ELMOD3, ENTPD5, FAM119A, FAM179A, CLIC2, COX7A2L, CREB3L4, CSF1, CSNK2A2, CSTA, CSTB, CTPS, CTSD, FST, GATA2* |
|  | CD56dim natural killer cell | *CYP27A1, DDX55, DYRK2, RPL37A, NOTCH3, AKR7A3, GPRC5C, GRIN1, HLA-E, PORCN, PSMC4, UPP1, IL21R, KIR2DS1, KIR2DS2, KIR2DS5* |
|  | Myeloid derived suppressor cell | *CCR2, CD14, CD2, CD86, CXCR4, FCGR2A, FCGR2B, FCGR3A, FERMT3, GPSM3, IL18BP, IL4R, ITGAL, ITGAM, PARVG, PSAP, PTGER2, PTGES2, S100A8, S100A9* |
|  | Natural killer T cell | *BTN2A2, CD101, CD109, CNPY3, CNPY4, CREB1, CRTC2, CRTC3, CSF2, KLRC1, FUT4, ICAM2, IL32, LAMP2, LILRB5, KLRG1, HSPA4, HSPB6, ISM2, ITIH2, KDM4C, KIR2DS4, KIRREL3, SDCBP, NFATC2IP, MICB* |
|  | Activated dendritic cell | *ABCD1, C1QC, CAPG, CCL3L3, CD207, CD302, ATP5B, ATP5L, ATP6V1A, BCL2L1, C1QB, SNURF, SPCS3, CCNA1, CEACAM8, NOS2, SRA1, TNFRSF6B, TREM1, TREML1, RHOA, SLC25A37, TNFSF14, TREML4, VNN2, XPO6* |
|  | Plasmacytoid dendritic cell | *CBX6, DAB2, DDX17, HIGD1A, IDH3A, IL3RA, MAGED1, NUCB2, OFD1, OGT, PDIA4, SERTAD2, SIRPA, TMED2, ENG, FCAR, IGF1, ITGA2B, GABARAP, GPX1, KRT23, PROK2, RALB, RETNLB, RNF141, SEC14L1* |
|  | Immature dendritic cell | *ACADM, AHCYL1, ALDH1A2, ALDH3A2, ALDH9A1, ALOX15, AMT, ARL1, ATIC, ATP5A1, CAPZA1, LILRA5, RDX, RRAGD, TACSTD2, INPP5F, RAB38, PLAU, CSF3R, SLC18A2, AMPD2, CLTB, C1orf162* |
|  | Macrophage | *AIF1, CCL1, CCL14, CCL23, CCL26, CD300LB, CNR1, CNR2, EIF1, EIF4A1, FPR1, FPR2, FRAT2, GPR27, GPR77, RNASE2, MS4A2, BASP1, IGSF6, HK3, VNN1, FES, NPL, FZD2, FAM198B, HNMT* |
|  | Eosinophil | *GIPR, KRT18P50, LRMP, FOSB, RRP12, GPR183, NR4A3, ST3GAL6, DEPDC5, PDE6C, PKD2L2, GPR65, IL5RA, P2RY14, DACH1, DAPK2, EMR3* |
|  | Mast cell | *ADAMTS3, CPA3, CMA1, CTSG, ARHGAP15, CPM, FCN1, FTL, HSPA6, ITGA9, RNASE3, S100A4, SIGLEC8, SLC6A4, PTGS2, EGR3, PILRA* |
|  | Monocyte | *ASGR2, CFP, ASGR1, CD1D, UPK3A, ACTG1, ANXA5, ATP6V1B2, CFL1, DAZAP2, CTBS, EMR4P, HIVEP2, MARCKSL1, MBP, MMP15, PNPLA6, TMBIM6, PQBP1, TEX264, IKZF1* |
|  | Neutrophil | *CREB5, CDA, CHST15, S100A12, APOBEC3A, CASP5, MMP25, HAL, C1orf183, FFAR2, MAK, CXCR1, STEAP4, MGAM, BTNL8, CXCR2, TNFRSF10C, VNN3* |

| **Oncogenic pathways** | PI3K-Akt signaling | *EGF, TGFA, EREG, AREG, FGF1, FGF2, FGF3, FGF4, FGF17, FGF6, FGF7, FGF8, FGF9, FGF10, FGF16, FGF5, FGF18, FGF20, FGF22, FGF19, FGF21, FGF23, NGF, BDNF, NTF3, NTF4, INS, IGF1, IGF2, PDGFA, PDGFB, PDGFC, PDGFD, CSF1, KITLG, FLT3LG, VEGFA, VEGFB, PGF, VEGFC, VEGFD, HGF, ANGPT1, ANGPT2, ANGPT4, EFNA1, EFNA2, EFNA3, EFNA4, EFNA5, EGFR, ERBB2, ERBB3, ERBB4, FGFR1, FGFR2, FGFR3, FGFR4, NGFR, NTRK1, NTRK2, INSR, IGF1R, PDGFRA, PDGFRB, CSF1R, KIT, FLT3, FLT1, FLT4, KDR, MET, TEK, EPHA2, GRB2, SOS1, SOS2, HRAS, KRAS, NRAS, RAF1, MAP2K1, MAP2K2, MAPK1, MAPK3, IRS1, TLR2, TLR4, RAC1, IGH, SYK, CD19, PIK3AP1, GH1, GH2, CSH1, CSH2, PRL, OSM, IL2, IL3, IL6, IL4, IL7, IFNA1, IFNA2, IFNA4, IFNA5, IFNA6, IFNA7, IFNA8, IFNA10, IFNA13, IFNA14, IFNA16, IFNA17, IFNA21, IFNB1, EPO, CSF3, GHR, PRLR, OSMR, IL2RA, IL2RB, IL2RG, IL3RA, IL6R, IL4R, IL7R, IFNAR1, IFNAR2, EPOR, CSF3R, JAK1, JAK2, JAK3, COL1A1, COL1A2, COL2A1, COL4A2, COL4A4, COL4A6, COL4A1, COL4A5, COL4A3, COL6A1, COL6A2, COL6A3, COL6A6, COL6A5, COL9A1, COL9A2, COL9A3, LAMA1, LAMA2, LAMA3, LAMA5, LAMA4, LAMB1, LAMB2, LAMB3, LAMB4, LAMC1, LAMC2, LAMC3, CHAD, RELN, THBS1, COMP, THBS2, THBS3, THBS4, FN1, SPP1, VTN, TNC, TNN, TNR, TNXB, VWF, IBSP, ITGA1, ITGA2, ITGA2B, ITGA3, ITGA4, ITGA5, ITGA6, ITGA7, ITGA8, ITGA9, ITGA10, ITGA11, ITGAV, ITGB1, ITGB3, ITGB4, ITGB5, ITGB6, ITGB7, ITGB8, PTK2, PIK3CA, PIK3CD, PIK3CB, PIK3R1, PIK3R2, PIK3R3, F2R, CHRM1, CHRM2, LPAR1, LPAR2, LPAR3, LPAR4, LPAR5, LPAR6, GNB1, GNB2, GNB3, GNB4, GNB5, GNG2, GNG3, GNG4, GNG5, GNG7, GNG8, GNG10, GNG11, GNG12, GNG13, GNGT1, GNGT2, PIK3CG, PIK3R5, PIK3R6, PDPK1, STK11, PRKAA1, PRKAA2, DDIT4, TSC1, TSC2, RHEB, MLST8, MTOR, RPTOR, EIF4EBP1, EIF4E, EIF4E2, EIF4E1B, RPS6KB1, RPS6KB2, EIF4B, RPS6, PRKCA, PKN1, PKN2, PKN3, SGK1, SGK2, SGK3, C8orf44-SGK3, AKT1, AKT2, AKT3, MAGI1, MAGI2, PTEN, THEM4, PPP2CA, PPP2CB, PPP2R1B, PPP2R1A, PPP2R2A, PPP2R2B, PPP2R2C, PPP2R2D, PPP2R3B, PPP2R3C, PPP2R3A, PPP2R5B, PPP2R5C, PPP2R5D, PPP2R5E, PPP2R5A, HSP90AA1, HSP90AB1, HSP90B1, CDC37, CRTC2, PHLPP1, PHLPP2, TCL1A, TCL1B, MTCP1, NOS3, BRCA1, GSK3B, GYS2, GYS1, PCK1, PCK2, G6PC, G6PC2, G6PC3, MYC, CCND1, CDKN1A, CDKN1B, CDK2, CDK4, CDK6, CCND2, CCND3, CCNE1, CCNE2, FOXO3, RBL2, FASLG, BCL2L11, YWHAZ, YWHAB, YWHAQ, YWHAE, YWHAH, YWHAG, BAD, BCL2L1, BCL2, CASP9, CREB1, ATF2, ATF4, CREB3, CREB3L1, CREB3L2, CREB3L3, CREB3L4, CREB5, ATF6B, MCL1, RXRA, NR4A1, IKBKG, CHUK, IKBKB, RELA, NFKB1, MYB, MDM2, TP53* |
| --- | --- | --- |
|  | VEGF signaling | *VEGFA, KDR, SH2D2A, PLCG1, PLCG2, PRKCA, PRKCB, PRKCG, SPHK1, SPHK2, HRAS, KRAS, NRAS, RAF1, MAP2K1, MAP2K2, MAPK1, MAPK3, PLA2G4E, PLA2G4A, JMJD7-PLA2G4B, PLA2G4B, PLA2G4C, PLA2G4D, PLA2G4F, PPP3CA, PPP3CB, PPP3CC, PPP3R1, PPP3R2, NFATC2, PTGS2, PTK2, SHC2, PXN, CDC42, MAPK11, MAPK12, MAPK13, MAPK14, MAPKAPK2, MAPKAPK3, HSPB1, SRC, PIK3CA, PIK3CD, PIK3CB, PIK3R1, PIK3R2, PIK3R3, RAC1, RAC2, RAC3, AKT1, AKT2, AKT3, NOS3, CASP9, BAD* |
|  | MAPK signaling | *CACNA1A, CACNA1B, CACNA1C, CACNA1D, CACNA1E, CACNA1F, CACNA1G, CACNA1H, CACNA1I, CACNA1S, CACNA2D1, CACNA2D2, CACNA2D3, CACNA2D4, CACNB1, CACNB2, CACNB3, CACNB4, CACNG1, CACNG2, CACNG3, CACNG4, CACNG5, CACNG6, CACNG7, CACNG8, PRKACA, PRKACB, PRKACG, PRKCA, PRKCB, PRKCG, GNA12, GNG12, PPP3CA, PPP3CB, PPP3CC, PPP3R1, PPP3R2, RASGRF1, RASGRF2, RASGRP1, RASGRP2, RASGRP3, RASGRP4, RAPGEF2, NF1, RASA1, RASA2, RAP1A, RAP1B, EGF, TGFA, EREG, AREG, FGF1, FGF2, FGF3, FGF4, FGF17, FGF6, FGF7, FGF8, FGF9, FGF10, FGF16, FGF5, FGF18, FGF20, FGF22, FGF19, FGF21, FGF23, NGF, BDNF, NTF3, NTF4, INS, IGF1, IGF2, PDGFA, PDGFB, PDGFC, PDGFD, CSF1, KITLG, FLT3LG, VEGFA, VEGFB, PGF, VEGFC, VEGFD, HGF, ANGPT1, ANGPT2, ANGPT4, EFNA1, EFNA2, EFNA3, EFNA4, EFNA5, EGFR, ERBB2, ERBB3, ERBB4, FGFR1, FGFR2, FGFR3, FGFR4, NGFR, NTRK1, NTRK2, INSR, IGF1R, PDGFRA, PDGFRB, CSF1R, KIT, FLT3, FLT1, FLT4, KDR, MET, TEK, EPHA2, GRB2, SOS1, SOS2, HRAS, KRAS, NRAS, RRAS, RRAS2, MRAS, ARAF, BRAF, RAF1, MAP2K1, MAP2K2, LAMTOR3, MAPK1, MAPK3, MKNK1, MKNK2, RPS6KA3, RPS6KA1, RPS6KA2, RPS6KA6, ATF4, ELK1, ELK4, MYC, SRF, FOS, MAPT, STMN1, PLA2G4E, PLA2G4A, JMJD7-PLA2G4B, PLA2G4B, PLA2G4C, PLA2G4D, PLA2G4F, TNF, IL1A, IL1B, TGFB1, TGFB2, TGFB3, TNFRSF1A, IL1R1, IL1RAP, TGFBR1, TGFBR2, FASLG, FAS, CD14, RAC1, RAC2, RAC3, CDC42, TRADD, CASP3, TRAF2, DAXX, MYD88, IRAK1, IRAK4, TRAF6, GADD45A, GADD45B, GADD45G, TAB1, TAB2, ECSIT, MAP4K3, MAP4K4, MAP4K1, PAK1, PAK2, STK4, STK3, MAP4K2, MAP3K8, MAP3K1, MAP3K11, MAP3K2, MAP3K3, MAP3K13, MAP3K12, MAP3K20, MAP3K6, MAP3K5, MAP3K7, MAP3K4, TAOK2, TAOK3, TAOK1, MAP2K4, MAP2K7, MAP2K3, MAP2K6, MAPK8IP1, MAPK8IP2, MAPK8IP3, FLNA, FLNC, FLNB, CRK, CRKL, ARRB1, ARRB2, MAPK8, MAPK10, MAPK9, MAPK11, MAPK12, MAPK13, MAPK14, MAPKAPK5, MAPKAPK2, MAPKAPK3, RPS6KA5, RPS6KA4, CDC25B, NFATC1, NFATC3, JUN, JUND, ATF2, TP53, DDIT3, MAX, MEF2C, HSPB1, AKT1, AKT2, AKT3, PPM1A, PTPRR, PTPN5, PTPN7, DUSP1, DUSP4, DUSP2, DUSP7, DUSP8, DUSP5, DUSP16, DUSP6, DUSP9, DUSP10, DUSP3, PPP5C, PPM1B, HSPA8, HSPA1A, HSPA2, HSPA1L, HSPA1B, HSPA6, MECOM, MAP2K5, MAPK7, NR4A1, MAP3K14, CHUK, IKBKB, IKBKG, NLK, NFKB1, NFKB2, RELA, RELB* |
|  | JAK-STAT signaling | *IL2, IL3, IL4, IL5, IL6, IL7, IL9, IL10, IL11, IL12A, IL12B, IL13, IL15, IL17D, IL19, IL20, IL21, IL22, IL23A, IL24, IFNA1, IFNA2, IFNA4, IFNA5, IFNA6, IFNA7, IFNA8, IFNA10, IFNA13, IFNA14, IFNA16, IFNA17, IFNA21, IFNB1, IFNG, IFNE, IFNK, IFNL1, IFNL2, IFNL3, IFNW1, OSM, LIF, TSLP, CTF1, CSF2, CNTF, CSF3, EPO, GH1, GH2, CSH1, CSH2, LEP, THPO, PRL, EGF, PDGFA, PDGFB, IL2RA, IL2RB, IL2RG, IL3RA, IL4R, IL5RA, IL6R, IL7R, IL9R, IL10RA, IL10RB, IL11RA, IL12RB1, IL12RB2, IL13RA1, IL13RA2, IL15RA, IL20RA, IL20RB, IL21R, IL22RA1, IL22RA2, IL23R, IL27RA, IL6ST, IFNAR1, IFNAR2, IFNGR1, IFNGR2, IFNLR1, OSMR, LIFR, CRLF2, CNTFR, CSF2RA, CSF2RB, CSF3R, EPOR, GHR, LEPR, MPL, PRLR, EGFR, PDGFRA, PDGFRB, JAK1, JAK2, JAK3, TYK2, STAT1, STAT2, STAT3, STAT4, STAT5A, STAT5B, STAT6, CISH, SOCS1, SOCS2, SOCS3, SOCS4, SOCS5, SOCS7, SOCS6, BCL2, MCL1, BCL2L1, PIM1, MYC, CCND1, CCND2, CCND3, CDKN1A, AOX1, GFAP, STAM2, STAM, PTPN2, PTPN6, IRF9, CREBBP, EP300, PIAS1, PIAS2, PIAS3, PIAS4, FHL1, PTPN11, GRB2, SOS1, SOS2, HRAS, RAF1, PIK3CA, PIK3CD, PIK3CB, PIK3R1, PIK3R2, PIK3R3, AKT1, AKT2, AKT3, MTOR* |
|  | Ras signaling | *EGF, TGFA, FGF1, FGF2, FGF3, FGF4, FGF17, FGF6, FGF7, FGF8, FGF9, FGF10, FGF16, FGF5, FGF18, FGF20, FGF22, FGF19, FGF21, FGF23, NGF, BDNF, NTF3, NTF4, INS, IGF1, IGF2, PDGFA, PDGFB, PDGFC, PDGFD, CSF1, KITLG, FLT3LG, VEGFA, VEGFB, PGF, VEGFC, VEGFD, HGF, ANGPT1, ANGPT2, ANGPT4, EFNA1, EFNA2, EFNA3, EFNA4, EFNA5, EGFR, FGFR1, FGFR2, FGFR3, FGFR4, NGFR, NTRK1, NTRK2, INSR, IGF1R, PDGFRA, PDGFRB, CSF1R, KIT, FLT3, FLT1, FLT4, KDR, MET, TEK, EPHA2, GRB2, GAB1, GAB2, SHC1, SHC2, SHC3, SHC4, PTPN11, SOS1, SOS2, PLCG1, PLCG2, RASGRP1, RASGRP2, RASGRP3, RASGRP4, ZAP70, LAT, HTR7, GNB1, GNB2, GNB3, GNB4, GNB5, GNG2, GNG3, GNG4, GNG5, GNG7, GNG8, GNG10, GNG11, GNG12, GNG13, GNGT1, GNGT2, PRKACA, PRKACB, PRKACG, RASGRF1, RASGRF2, GRIN1, GRIN2A, GRIN2B, CALML3, CALM2, CALM3, CALM1, CALML6, CALML5, CALML4, HRAS, KRAS, NRAS, MRAS, RRAS, RRAS2, NF1, RASA1, RASA2, RASA3, RASA4, RASA4B, SYNGAP1, RASAL1, RASAL2, RASAL3, RASSF1, RASSF5, STK4, TIAM1, RAC1, RAC2, RAC3, PAK1, PAK2, PAK3, PAK4, PAK5, PAK6, BUB1B-PAK6, RHOA, PIK3CA, PIK3CD, PIK3CB, PIK3R1, PIK3R2, PIK3R3, AKT1, AKT2, AKT3, IKBKG, CHUK, IKBKB, NFKB1, RELA, BAD, BCL2L1, FOXO4, FASLG, AFDN, SHOC2, RAF1, MAP2K1, MAP2K2, MAPK1, MAPK3, PLA1A, PLA2G10, PLA2G2D, PLA2G2E, PLA2G3, PLA2G2F, PLA2G12A, PLA2G12B, PLA2G1B, PLA2G5, PLA2G2A, PLA2G2C, PLA2G4E, PLA2G4A, JMJD7-PLA2G4B, PLA2G4B, PLA2G4C, PLA2G4D, PLA2G4F, PLA2G6, PLAAT3, ELK1, ETS1, ETS2, BRAP, KSR1, KSR2, RAPGEF5, RAP1A, RAP1B, RALGDS, RGL1, RGL2, RALA, RALB, MAPK8, MAPK10, MAPK9, EXOC2, TBK1, REL, PLD1, PLD2, RALBP1, CDC42, PLCE1, PRKCA, PRKCB, PRKCG, RIN1, ABL1, ABL2, RAB5A, RAB5B, RAB5C, ARF6* |
|  | HIF-1 signaling | *IL6, IL6R, STAT3, TLR4, IFNG, IFNGR1, IFNGR2, RELA, NFKB1, INS, EGF, IGF1, INSR, EGFR, IGF1R, ERBB2, MAP2K1, MAP2K2, MAPK1, MAPK3, MKNK1, MKNK2, PIK3CA, PIK3CD, PIK3CB, PIK3R1, PIK3R2, PIK3R3, AKT1, AKT2, AKT3, MTOR, EIF4EBP1, EIF4E, EIF4E2, EIF4E1B, RPS6KB1, RPS6KB2, RPS6, HIF1A, VHL, RBX1, ELOC, ELOB, CUL2, EGLN1, EGLN3, EGLN2, ARNT, CREBBP, EP300, CYBB, PLCG1, PLCG2, PRKCA, PRKCB, PRKCG, CAMK2A, CAMK2D, CAMK2B, CAMK2G, TIMP1, LTBR, EPO, TF, TFRC, VEGFA, FLT1, SERPINE1, ANGPT1, ANGPT2, ANGPT4, TEK, EDN1, NOS2, NOS3, HMOX1, NPPA, SLC2A1, PDK1, HK3, HK1, HK2, HKDC1, PFKM, PFKP, PFKL, GAPDH, ALDOC, ALDOA, ALDOB, ENO3, ENO2, ENO1, ENO4, PGK2, PGK1, PFKFB3, LDHAL6A, LDHAL6B, LDHA, LDHB, LDHC, BCL2, CDKN1A, CDKN1B, PDHA2, PDHA1, PDHB* |

| **DNA damage repair (DDR) pathways** | Base excision repair (BER) | *PARP1, POLB, APEX1, APEX2, FEN1, TDG, TDP1, UNG* |
| --- | --- | --- |
|  | Nucleotide excision repair (NER) | *CUL5, ERCC1, ERCC2, ERCC4, ERCC5, ERCC6, POLE, POLE3, XPA, XPC* |
|  | Mismatch repair (MMR) | *EXO1, MLH1, MLH3, MSH2, MSH3, MSH6, PMS1, PMS2* |
|  | Fanconi anemia (FA) | *FANCA, FANCB, FANCC, FANCD2, FANCI, FANCL, FANCM, UBE2T* |
|  | Homologous recomination (HR) | *MRE11A, NBN, RAD50, TP53BP1, XRCC2, XRCC3, BARD1, BLM, BRCA1, BRCA2, BRIP1, EME1, GEN1, MUS81, PALB2, RAD51, RAD52, RBBP8, SHFM1, SLX1A, TOP3A* |
|  | Non-homologous end joining (NHEJ) | *LIG4, NHEJ1, POLL, POLM, PRKDC, XRCC4, XRCC5, XRCC6* |
|  | Direct damage reversal/repair (DR) | *ALKBH2, ALKBH3, MGMT* |
|  | Translesion DNA synthesis (TLS) | *POLN, POLQ, REV1, REV3L, SHPRH* |
|  | Damage sensor (DS) | *ATM, ATR, ATRIP, CHEK1, CHEK2, MDC1, RNMT, TOPBP1, TREX1* |
| **Biological processes** | EMT | *CDH2, SNAI1, TGFB1, ZEB1, ZEB2* |
|  | Invasion | *AEBP1, AKR1B1, AMD1, SLC25A5, ATP5PB, BAG1, BGN, C1QB, CALD1, CAPG, CCNE1, CDH11, CKS1B, CKS2, COL1A1, COL1A2, COL3A1, COL5A1, COL5A2, COL6A2, COL6A3, COL10A1, COL11A1, COMP, CSE1L, VCAN, CTSK, DAB2, DDX5, EDNRA, FAP, FBN1, FN1, GNAS, H2AFZ, HMGB2, HNRNPU, HSD17B4, CYR61, INHBA, LAMB1, LAMC1, LGALS1, LOX, LOXL2, LUM, MMP2, MMP11, HNRNPM, NDUFB7, YBX1, PDGFRB, PLAU, PRRX1, PNN, PPIC, PROS1, PSMA2, PSMB4, RGS4, SNAI2, SPOCK1, TGFBI, THBS2, THY1, TNFAIP6, UBE2V2, ADAM12, MFAP5, ITGBL1, TP53I3, NUAK1, HNRNPDL, TXNDC9, LRRC17, IFI30, POSTN, CBX1, NID2, RRAS2, RALY, SEPHS2, HEY1, MXRA5, OLFML2B, TMEM158, WWTR1, GREM1, NOX4, CLEC4A, COPZ2, ASPN, CEMIP, CRISPLD2, TUBB6, LRRC15, TUBB* |
|  | Metastasis | *ACKR3, AFP, AGR2, AKT1, ALDH1A1, ANGPTL4, ANXA1, ANXA2, AQP5, ATF3, AURKA, AXL, B2M, BMI1, BMP2, BRAF, BSG, CA9, CCDC88A, CCND1, CCR7, CD24, CD274, CD44, CDCP1, CDH17, CDH2, CEACAM1, CEACAM5, CLIC1, CRP, CTGF, CTNND1, CTSB, CTSK, CTSL, CTTN, CXCL12, CXCL2, CXCL8, CXCR2, CYR61, DDR2, EGF, EGFR, EGR1, EPAS1, EPCAM, ERBB2, ERBB3, ESR1, ESR2, EZH2, EZR, F2RL1, FGFR1, FLOT2, FLT1, FLT4, FN1, FOXC2, FOXM1, GLI1, HGF, HIF1A, HMGA1, HMGA2, HPSE, ID1, IDO1, IGF1R, IGF2BP3, IL6, ITGA3, ITGA5, ITGA6, ITGAV, ITGB1, ITGB3, JAG1, KDR, KRAS, KRT19, L1CAM, LAMC2, LASP1, LGALS3, LOX, LOXL2, MACC1, MCAM, MDM2, MET, MIF, MKI67, MME, MMP1* |
|  | Proliferation | *ADK, BAX, CCND1, BMPR1A, BUB1B, CCNA2, CCNB1, CD40, CDK1, CDC20, CYBA, HMGB2, IGFBP2, MKI67, PRKD1, TEK, TK1, TOP2A, DLGAP5, MELK, GINS1, KIF14, RACGAP1, ASPM, CD24, CX3CL1, IGF1, CD74, TP63, FGFR1, DCT, PTPRC, LHX5, ESR1, ABL1, XBP1, PDGFB, HIF1A, CD40LG, FGF9, FZD3, RIPK2, NUMBL, EBI3, WNT2, IL2, IL23A, VEGFA, SEMA5A, IL4, CD86, STAT1, ID2, DOCK7, SLAMF1, TNFSF4, WNT1, TNFSF9, MED1, SMO, TLR4, MYC, LEF1, ASCL1, FGF7, OSR1, IL18, WNT3A, WNT7A, DISC1, BMP10, SYK, IL13, BCL2, ID4, STAT5B, CD34, CTC1, TBX1, IRS2, TNFRSF4, HMGB1, ZNF335, NRARP, BMPR2, AGER, MIF, CCL5* |
|  | Proliferation | *CCNB1, CDC20, CDKN3, CDK1, MAD2L1, PRC1, RRM2* |
| **Immune-related pathways for clustering cancer cells** | Antigen processing and presentation | *IFNG, TNF, PSME1, PSME2, PSME3, HSPA8, HSPA1A, HSPA2, HSPA1L, HSPA1B, HSPA6, HSPA4, HSP90AA1, HSP90AB1, HLA-A, HLA-B, HLA-C, HLA-F, HLA-G, HLA-E, HSPA5, CANX, B2M, PDIA3, CALR, TAPBP, TAP1, TAP2, CD8A, CD8B, CD8B2, KIR3DL2, KIR3DL1, KIR3DL3, KIR2DL2, KIR2DL1, KIR2DL3, KIR2DL4, KIR2DL5A, KLRC1, KLRC2, KLRC3, KLRC4, KLRD1, KIR2DS1, KIR2DS3, KIR2DS4, KIR2DS5, KIR2DS2, IFI30, LGMN, CTSB, HLA-DMA, HLA-DMB, HLA-DOA, HLA-DOB, HLA-DPA1, HLA-DPB1, HLA-DQA1, HLA-DQA2, HLA-DQB1, HLA-DRA, HLA-DRB1, HLA-DRB3, HLA-DRB4, HLA-DRB5, CD74, CTSL, CTSS, CD4, CIITA, RFX5, RFXANK, RFXAP, CREB1, NFYA, NFYB, NFYC* |
|  | PD-L1 expression pathway in cancer | *MAPK1, EGF, EGFR, HIF1A, HRAS, RAF1, MAP2K1, FOS, PTEN, ALK, PIK3CA, AKT3, MTOR, RPS6KB1, CHUK, IFNG, IFNGR1, JAK1, STAT1, TIRAP, TRAF6, NFATC1, TLR9, TICAM2, NFKBIA, NFKB1, CD274, MYD88, TICAM1, ERK, ERK-2, ERK2, ERT1, MAPK2, NS13, P42MAPK, PRKM1, PRKM2, p38, p40, p41, p41mapk, p42-MAPK, HOMG4, URG, ERBB, ERBB1, ERRP, HER1, NISBD2, PIG61, mENA, HIF-1-alpha, HIF-1A, HIF-1alpha, HIF1, HIF1-ALPHA, MOP1, PASD8, bHLHe78, C-BAS/HAS, C-H-RAS, C-HA-RAS1, CTLO, H-RASIDX, HAMSV, HRAS1, RASH1, p21ras, CMD1NN, CRAF, NS5, Raf-1, c-Raf, CFC3, MAPKK1, MEK1, MEL, MKK1, PRKMK1, AP-1, C-FOS, p55, 10q23del, BZS, CWS1, DEC, GLM2, MHAM, MMAC1, PTEN1, PTENbeta, TEP1, FRAP2, RAFT1, RAPT1, SKS, NBLST3, CD246, CLAPO, MPPH, FRAP, PS6K, IKBKA, IFG, CD119, AIIDE, CANDF7, BACTS1, MGC:3310, NF-ATC, CD289, MyD88-4, EDAID2, CVID12, B7-H, IMD68, IIAE6, CLOVE, CWS5, MCAP, MCM, MCMTC, PI3K, PI3K-alpha, p110-alpha, MPPH2, FRAP1, S6K, IKK-alpha, IFI, IFNGR, JAK1A, IMD31A, Mal, RNF85, NF-ATc1.2, TICAM-2, IKBA, EBP-1, B7H1, MYD88D, MyD88-3, PKB-GAMMA, PKBG, PRKBG, RAC-PK-gamma, RAC-gamma, STK-2, S6K-beta-1, S6K1, STK14A, p70_S6KA, p70(S6K)-alpha, p70-S6K, p70-alpha, IKK1, IMD69, IMD27A, JAK1B, IMD31B, MyD88-2, NFAT2, STAT91, TIRAP3, MAD-3, KBF1, PD-L1, NF-kappabeta, PRVTIRB, NFKB-p105, IKKA, TCF16, IMD27B, JTK3, IMD31C, wyatt, NFKBIKA, NFATc, ISGF-3, TIRP, NFKBI, NF-kB, PDCD1L1, NF-kappaB, TICAM-1, NFKB-p50, NFkappaB, TRAM, NF-kappa-B1, NF-kB1, PDCD1LG1, PDL1, TRIF, hPD-L1* |
|  | Apoptosis | *TNFSF10, TNFRSF10A, TNFRSF10B, FASLG, FAS, FADD, TNF, TNFRSF1A, TRADD, CFLAR, CASP8, CASP10, CASP6, CASP3, CASP7, BID, BAX, BAK1, DIABLO, SEPTIN4, HTRA2, CYCS, APAF1, CASP9, PRF1, GZMB, TUBA1B, TUBA4A, TUBA3C, TUBA1A, TUBA1C, TUBA8, TUBA3E, TUBA3D, TUBAL3, MCL1, ACTG1, ACTB, SPTA1, SPTAN1, LMNA, LMNB1, LMNB2, PARP1, PARP2, PARP3, PARP4, DFFA, DFFB, ENDOG, AIFM1, ERN1, TRAF2, ITPR1, ITPR2, ITPR3, CAPN1, CAPN2, CASP12, EIF2AK3, EIF2S1, ATF4, DDIT3, CTSB, CTSC, CTSD, CTSF, CTSH, CTSK, CTSL, CTSO, CTSS, CTSV, CTSW, CTSZ, BIRC2, BIRC3, XIAP, BIRC5, BCL2L11, BCL2L1, BCL2, DAXX, RIPK1, DAB2IP, MAP3K5, MAPK8, MAPK10, MAPK9, BAD, JUN, FOS, TP53, HRK, MAP3K14, CHUK, IKBKB, IKBKG, NFKBIA, NFKB1, RELA, PTPN13, GADD45A, GADD45B, GADD45G, TRAF1, BCL2A1, ATM, PIDD1, TP53AIP1, BBC3, PMAIP1, CASP2, NGF, NTRK1, IL3, IL3RA, CSF2RB, PIK3CA, PIK3CD, PIK3CB, PIK3R1, PIK3R2, PIK3R3, PDPK1, AKT1, AKT2, AKT3, HRAS, KRAS, NRAS, RAF1, MAP2K1, MAP2K2, MAPK1, MAPK3* |
|  | JAK-STAT signaling | *IL2, IL3, IL4, IL5, IL6, IL7, IL9, IL10, IL11, IL12A, IL12B, IL13, IL15, IL17D, IL19, IL20, IL21, IL22, IL23A, IL24, IFNA1, IFNA2, IFNA4, IFNA5, IFNA6, IFNA7, IFNA8, IFNA10, IFNA13, IFNA14, IFNA16, IFNA17, IFNA21, IFNB1, IFNG, IFNE, IFNK, IFNL1, IFNL2, IFNL3, IFNW1, OSM, LIF, TSLP, CTF1, CSF2, CNTF, CSF3, EPO, GH1, GH2, CSH1, CSH2, LEP, THPO, PRL, EGF, PDGFA, PDGFB, IL2RA, IL2RB, IL2RG, IL3RA, IL4R, IL5RA, IL6R, IL7R, IL9R, IL10RA, IL10RB, IL11RA, IL12RB1, IL12RB2, IL13RA1, IL13RA2, IL15RA, IL20RA, IL20RB, IL21R, IL22RA1, IL22RA2, IL23R, IL27RA, IL6ST, IFNAR1, IFNAR2, IFNGR1, IFNGR2, IFNLR1, OSMR, LIFR, CRLF2, CNTFR, CSF2RA, CSF2RB, CSF3R, EPOR, GHR, LEPR, MPL, PRLR, EGFR, PDGFRA, PDGFRB, JAK1, JAK2, JAK3, TYK2, STAT1, STAT2, STAT3, STAT4, STAT5A, STAT5B, STAT6, CISH, SOCS1, SOCS2, SOCS3, SOCS4, SOCS5, SOCS7, SOCS6, BCL2, MCL1, BCL2L1, PIM1, MYC, CCND1, CCND2, CCND3, CDKN1A, AOX1, GFAP, STAM2, STAM, PTPN2, PTPN6, IRF9, CREBBP, EP300, PIAS1, PIAS2, PIAS3, PIAS4, FHL1, PTPN11, GRB2, SOS1, SOS2, HRAS, RAF1, PIK3CA, PIK3CD, PIK3CB, PIK3R1, PIK3R2, PIK3R3, AKT1, AKT2, AKT3, MTOR* |
| **T cell subpopulations** | CD4+ FOXP3for regulatory CD4+ T cell | *FOXP3, CTLA4, IL2RA* |
|  | CD4+ CXCL13for activated CD4+ T cell | *CXCL13, CD200, ICOS* |
|  | CD4+ IL7Rfor resting CD4+ T cell | *IL7R, GPR183, LMNA, ANXA1* |
|  | CD8 + GZMB T cell | *ENTPD1, CXCL13* |

| **Table S3. Genes with significantly different mutation rates between the immune subtypes of MSI-L/MSS COAD in TCGA-COAD** | | | | | | | | | | | |
| --- | --- | --- | --- | --- | --- | --- | --- | --- | --- | --- | --- |
| **IM-H vs. IM-L** | | | | | **Immune score in**  **mutated vs. wildtype** | | **IM-M vs. IM-H/L** | | | | |
| **Gene** | ***P*-value**  **(Fisher’s exact test)** | **Odds ratio** | **Mutation rate**  **in IM-H** | **Mutation rate**  **in IM-L** | **Gene** | ***P*-value**  **(one-tailed Mann–Whitney U test)** | **Gene** | ***P*-value**  **(Fisher’s exact test)** | **Odds ratio** | **Mutation rate**  **in IM-M** | **Mutation rate**  **in IM-H/L** |
| *CHD5* | 0.0157 | 0 | 0.00% | 11.29% | *CHD5* | 0.9740 | *CUBN* | 0.0367 | 0.1409 | 1.79% | 11.50% |
| *DCLK1* | 0.0315 | 0 | 0.00% | 9.68% | *DCLK1* | 0.6016 | *ATXN1* | 0.0349 | 2.3768 | 26.79% | 13.27% |
| *FBXL7* | 0.0315 | 0 | 0.00% | 9.68% | *FBXL7* | 0.9565 | *TMEM131* | 0.0401 | 3.3872 | 16.07% | 5.31% |
| *APC* | 0.0011 | 0.1367 | 72.55% | 95.16% | *APC* | 0.8029 | *ZNF707* | 0.0401 | 3.3872 | 16.07% | 5.31% |
| *COL6A6* | 0.0394 | 0.1369 | 1.96% | 12.90% | *COL6A6* | 0.9540 | *GPRIN2* | 0.0104 | 3.5492 | 21.43% | 7.08% |
| *KRTAP10-10* | 0.0394 | 0.1369 | 1.96% | 12.90% | *KRTAP10-10* | 0.9779 | *PPM1E* | 0.0104 | 3.5492 | 21.43% | 7.08% |
| *PCDHGA5* | 0.0394 | 0.1369 | 1.96% | 12.90% | *PCDHGA5* | 0.9678 | *ABCA7* | 0.0322 | 3.5695 | 14.29% | 4.42% |
| *USH2A* | 0.0330 | 3.4686 | 23.53% | 8.06% | *USH2A* | 0.0164 | *GRIN3B* | 0.0322 | 3.5695 | 14.29% | 4.42% |
| *HMCN1* | 0.0345 | 4.1621 | 17.65% | 4.84% | *HMCN1* | 0.0035 | *PRIC285* | 0.0038 | 3.8171 | 25.00% | 7.96% |
| *PTPRT* | 0.0410 | 5.5021 | 15.69% | 3.23% | *PTPRT* | 0.0121 | *KIF26A* | 0.0124 | 3.8420 | 17.86% | 5.31% |
| *ADAMTSL3* | 0.0446 | 8.0025 | 11.76% | 1.61% | *ADAMTSL3* | 0.0084 | *LAMA5* | 0.0124 | 3.8420 | 17.86% | 5.31% |
| *TDRD6* | 0.0446 | 8.0025 | 11.76% | 1.61% | *TDRD6* | 0.0028 | *KLHL30* | 0.0426 | 3.8585 | 12.50% | 3.54% |
| *TRO* | 0.0446 | 8.0025 | 11.76% | 1.61% | *TRO* | 0.0039 | *FREM2* | 0.0058 | 4.3164 | 19.64% | 5.31% |
| *TCHH* | 0.0219 | 9.5387 | 13.73% | 1.61% | *TCHH* | 0.0118 | *RECQL4* | 0.0212 | 4.4964 | 14.29% | 3.54% |
| *ATP8A2* | 0.0071 | Inf | 11.76% | 0.00% | *ATP8A2* | 0.0257 | *TMEM184A* | 0.0212 | 4.4964 | 14.29% | 3.54% |
| *CCDC9* | 0.0167 | Inf | 9.80% | 0.00% | *CCDC9* | 0.0187 | *NOTCH3* | 0.0105 | 5.1612 | 16.07% | 3.54% |
| *DCDC5* | 0.0388 | Inf | 7.84% | 0.00% | *DCDC5* | 0.0139 | *ADAMTS1* | 0.0161 | 5.1815 | 12.50% | 2.65% |
| *FADS3* | 0.0388 | Inf | 7.84% | 0.00% | *FADS3* | 0.0082 | *FIGNL2* | 0.0161 | 5.1815 | 12.50% | 2.65% |
| *LRRC7* | 0.0388 | Inf | 7.84% | 0.00% | *LRRC7* | 0.0019 | *MYH8* | 0.0161 | 5.1815 | 12.50% | 2.65% |
| *NOTCH3* | 0.0388 | Inf | 7.84% | 0.00% | *NOTCH3* | 0.0346 | *PCDHAC2* | 0.0161 | 5.1815 | 12.50% | 2.65% |
| *SPG20* | 0.0071 | Inf | 11.76% | 0.00% | *SPG20* | 0.0014 | *GPR124* | 0.0067 | 6.0384 | 14.29% | 2.65% |
|  |  |  |  |  |  |  | *PRSS36* | 0.0067 | 6.0384 | 14.29% | 2.65% |
|  |  |  |  |  |  |  | *POLRMT* | 0.0027 | 6.9316 | 16.07% | 2.65% |
|  |  |  |  |  |  |  | *ZAR1L* | 0.0066 | 7.8242 | 12.50% | 1.77% |
|  |  |  |  |  |  |  | *CYP2A7* | 0.0025 | 9.1200 | 14.29% | 1.77% |
|  |  |  |  |  |  |  | *SIPA1L3* | 0.0025 | 9.1200 | 14.29% | 1.77% |
|  |  |  |  |  |  |  | *UBXN11* | 0.0025 | 9.1200 | 14.29% | 1.77% |
|  |  |  |  |  |  |  | *SCARF2* | 0.0009 | 10.4696 | 16.07% | 1.77% |
|  |  |  |  |  |  |  | *SPHK1* | 0.0002 | 21.0734 | 16.07% | 0.88% |

| **Table S4. 93 proteins differentially expressed between IM-H and IM-L in TCGA-COAD** | | | |
| --- | --- | --- | --- |
| **Protein** | ***P*-value (Two-tailed Student’s *t* test)** | **log2FC** | **FDR** |
| Chk1_pS345 | 0.0169 | 0.0584 | 0.0407 |
| P-Cadherin | 0.0195 | 0.0591 | 0.0460 |
| Bid | 0.0037 | 0.0782 | 0.0113 |
| Mre11 | <0.0001 | 0.0898 | <0.0001 |
| CD20 | 0.0016 | 0.0924 | 0.0052 |
| CD31 | 0.0004 | 0.0931 | 0.0014 |
| p38_MAPK | 0.0085 | 0.0993 | 0.0220 |
| CHK1_pS296 | 0.0066 | 0.1048 | 0.0178 |
| Tuberin_pT1462 | 0.0042 | 0.1095 | 0.0125 |
| p27 | 0.0006 | 0.1297 | 0.0023 |
| ERCC1 | 0.0058 | 0.1348 | 0.0161 |
| ACVRL1 | 0.0015 | 0.1478 | 0.0050 |
| PREX1 | 0.0142 | 0.1522 | 0.0352 |
| Chk1 | 0.0001 | 0.1571 | 0.0004 |
| 14-3-3_zeta | 0.0091 | 0.1708 | 0.0233 |
| VEGFR2 | 0.0184 | 0.1715 | 0.0440 |
| Rab11 | <0.0001 | 0.1792 | <0.0001 |
| STAT3_pY705 | 0.0030 | 0.1819 | 0.0096 |
| FOXO3a_pS318_S321 | <0.0001 | 0.1904 | 0.0003 |
| YAP | 0.0002 | 0.1905 | 0.0010 |
| c-Kit | 0.0064 | 0.1906 | 0.0175 |
| G6PD | <0.0001 | 0.1962 | <0.0001 |
| MEK1 | 0.0001 | 0.2051 | 0.0005 |
| p21 | 0.0001 | 0.2224 | 0.0004 |
| N-Cadherin | 0.0002 | 0.2246 | 0.0008 |
| Lck | 0.0059 | 0.2279 | 0.0161 |
| TAZ | <0.0001 | 0.2282 | <0.0001 |
| Bcl-2 | 0.0001 | 0.2296 | 0.0003 |
| CD26 | 0.0001 | 0.2433 | 0.0007 |
| AR | <0.0001 | 0.2434 | 0.0001 |
| PEA15 | <0.0001 | 0.2725 | <0.0001 |
| Bcl2A1 | <0.0001 | 0.2754 | <0.0001 |
| Src_pY416 | <0.0001 | 0.2770 | <0.0001 |
| Transglutaminase | 0.0001 | 0.3164 | 0.0003 |
| Src_pY527 | 0.0011 | 0.3901 | 0.0038 |
| MAPK_pT202_Y204 | 0.0008 | 0.3907 | 0.0029 |
| Caveolin-1 | 0.0036 | 0.4711 | 0.0111 |
| PAI-1 | 0.0007 | 0.4735 | 0.0024 |
| Collagen_VI | <0.0001 | 0.5911 | <0.0001 |
| ETS-1 | 0.0006 | 0.6351 | 0.0023 |
| Annexin-1 | <0.0001 | 0.6453 | <0.0001 |
| Fibronectin | <0.0001 | 0.6791 | <0.0001 |
| Rictor | 0.0070 | 0.6935 | 0.0186 |
| HSP70 | <0.0001 | 0.8153 | <0.0001 |
| MYH11 | <0.0001 | 1.3326 | 0.0001 |
| beta-Catenin | <0.0001 | -0.9075 | <0.0001 |
| E-Cadherin | <0.0001 | -0.9074 | <0.0001 |
| Cyclin_B1 | <0.0001 | -0.7026 | <0.0001 |
| S6 | <0.0001 | -0.6371 | <0.0001 |
| FoxM1 | <0.0001 | -0.5005 | <0.0001 |
| ACC1 | <0.0001 | -0.4986 | <0.0001 |
| eIF4G | <0.0001 | -0.4960 | <0.0001 |
| FASN | 0.0003 | -0.4520 | 0.0012 |
| eEF2K | <0.0001 | -0.4508 | <0.0001 |
| MSH6 | <0.0001 | -0.4264 | <0.0001 |
| Bap1-c-4 | <0.0001 | -0.4082 | <0.0001 |
| 53BP1 | <0.0001 | -0.4074 | 0.0001 |
| RBM15 | <0.0001 | -0.3933 | <0.0001 |
| ACC_pS79 | <0.0001 | -0.3690 | 0.0002 |
| Claudin-7 | 0.0198 | -0.3685 | 0.0462 |
| Rb_pS807_S811 | 0.0042 | -0.3673 | 0.0126 |
| Ku80 | <0.0001 | -0.3487 | <0.0001 |
| BRD4 | <0.0001 | -0.3341 | 0.0001 |
| ERCC5 | <0.0001 | -0.3266 | <0.0001 |
| MSH2 | <0.0001 | -0.3228 | <0.0001 |
| TSC1 | <0.0001 | -0.3143 | <0.0001 |
| B-Raf | 0.0003 | -0.3048 | 0.0011 |
| COG3 | <0.0001 | -0.3026 | <0.0001 |
| p62-LCK-ligand | 0.0005 | -0.2824 | 0.0020 |
| Rab25 | 0.0056 | -0.2813 | 0.0158 |
| Chk2 | <0.0001 | -0.2807 | 0.0002 |
| AMPK_pT172 | 0.0006 | -0.2751 | 0.0023 |
| p90RSK | <0.0001 | -0.2735 | 0.0001 |
| 4E-BP1 | 0.0001 | -0.2626 | 0.0006 |
| CDK1_pY15 | <0.0001 | -0.2545 | <0.0001 |
| p70S6K | <0.0001 | -0.2384 | 0.0001 |
| HER3 | 0.0003 | -0.2375 | 0.0011 |
| Cyclin_E1 | 0.0019 | -0.1884 | 0.0061 |
| Tuberin | <0.0001 | -0.1882 | 0.0002 |
| Caspase-3 | 0.0001 | -0.1852 | 0.0005 |
| Smad1 | <0.0001 | -0.1838 | 0.0002 |
| Rad50 | 0.0056 | -0.1762 | 0.0159 |
| CD49b | 0.0045 | -0.1634 | 0.0130 |
| ARID1A | 0.0002 | -0.1502 | 0.0009 |
| Jak2 | 0.0003 | -0.1478 | 0.0014 |
| ADAR1 | 0.0137 | -0.1418 | 0.0346 |
| GATA3 | 0.0072 | -0.1364 | 0.0190 |
| mTOR | 0.0006 | -0.1298 | 0.0023 |
| Rb | 0.0010 | -0.1144 | 0.0034 |
| SF2 | 0.0142 | -0.1120 | 0.0355 |
| JAB1 | 0.0208 | -0.1101 | 0.0480 |
| FOXO3a | 0.0149 | -0.0997 | 0.0364 |
| PR | 0.0036 | -0.0563 | 0.0111 |
